# Construction and characterization of a high-quality cDNA library of *Cymbidium faberi* suitable for yeast one- and two-hybrid assays

**DOI:** 10.1186/s12896-020-0599-2

**Published:** 2020-01-16

**Authors:** Yanqin Xu, Junjiang Zhou, Qingqing Liu, Kunpeng Li, Yin Zhou

**Affiliations:** 1 College of Pharmacy, Jiangxi University of Traditional Chinese Medicine, Nanchang, 330006 People’s Republic of China; 2 Center of Applied Biotechnology, Wuhan University of Bioengineering, Wuhan, 430415 People’s Republic of China; 3 College of Bioscience and Biotechnology, Wuhan University of Bioengineering, Wuhan, 430415 People’s Republic of China; 4 Department of Protein Services, Wuhan Genecreate Bioengineering Co., Ltd, Wuhan, 430206 People’s Republic of China

**Keywords:** Yeast two hybrid, Yeast one hybrid, Three-frame cDNA library, Normalization, *Cymbidium faberi*

## Abstract

**Background:**

*Cymbidium faberi* is one of the oldest cultivars of oriental orchids, with an elegant flower fragrance. In order to investigate the molecular mechanism and the functions of related proteins in the methyl jasmonate (MeJA) signaling pathway, one of the main components of flower fragrance in *C. faberi*, yeast one- and two-hybrid three-frame cDNA libraries were constructed.

**Results:**

In this study, a modified cDNA library used for yeast one- and two-hybrid screening was successfully constructed, with a recombinant efficiency of 95%. The lengths of inserted fragments ranged from 750~3000 bp, and the library capacity reached 6 × 10^9^ CFU/ μg of cDNA insert, which was suitable for the requirements of subsequent screening. Finally, a homologous protein related with pathogenesis was screened out by the bait vector of CfbHLH36, which may participate in the MeJA signaling pathway.

**Conclusion:**

The yeast one- and two-hybrid library of *C. faberi* provides large amounts of useful information for the functional genomics research in *C. faberi*, and this method could also be applied to other plants to screen DNA-protein and protein-protein interactions.

## Background

Orchidaceae is one of the largest families of monocotyledonous plants. In cultivation, orchids are generally divided into tropical and oriental cultivars. *Cymbidium faberi* is famous for its soft color and strong flower fragrance. However, the wild populations have significantly deteriorated due to over-exploitation. In order to develop new cultivars of *Cymbidium* via genetic engineering and preserve the wild resources, it is imperative to elucidate the biosynthetic pathways and molecular mechanisms of its economically important traits.

The yeast one- and two-hybrid systems are commonly used to screen for interactions between target proteins and bait molecules. The yeast one-hybrid system is generally used to analyze DNA-protein interactions, while the yeast two-hybrid system can be used to analyze protein-protein interactions based on the expression of the reporter genes, and both are widely used in functional genomics studies [1]. In general, the yeast two-hybrid system requires a high-quality cDNA library. By contrast, there are two ways to construct a yeast one-hybrid library, the protein-centered approach and the DNA-centered approach. The former method requires a random short DNA sequence insertion library and the protein as the bait. The latter method requires a cDNA library and a cis-element as the bait [2]. Therefore, the cDNA library of the two-hybrid system can also be extended to yeast one-hybrid screening.

Based on the high-throughput screening and stringent screening pressure, many researches have obtained candidate prey proteins by yeast one- and two-hybrid assays, like in wheat [3, 4], Arabidopsis [5], rice [6], populus [7], kiwifruit [8] and so on. Moreover, increasing numbers of studies focused on improving the screening efficiency, saving time and decreasing the cost required to identify target molecules in yeast one- and two-hybrid system assays [9, 10].

Functional genomics based on transcriptomic profiling of the expression of different genes in different flowering stages was used to screen target proteins involved in the flower development of *C. faberi* [11]. Methyl jasmonate (MeJA) has been extensively explored as a volatile compound and a signal molecule to interact with other organisms (plants, animals and microbes) [12, 13]. The biosynthetic pathway of MeJA has been elaborated in model plants and its complicated regulation mechanism was continuously investigated [14]. Many transcription factors were demonstrated to be positive or negative regulators in MeJA metabolism, especially MYC gene family, like MYCs and JA-ASSOCIATED MYC2-LIKE (JAMs) [15], which could antagonistically regulate the downstream genes in MeJA signaling pathway.

In this study, we first constructed a cDNA library of *C. faberi* including almost all of the expressed genes in different tissues and different flowering stages, which extended the range of the subsequent screening of target proteins. Additionally, a yeast library was constructed and used for yeast hybrid assay, enabling the easy and fast screening of target proteins via bidirectional screening. The lengths of inserted fragments in the library ranged from 750~3000 bp, 65% of which were longer than 1500 bp. Due to the lack of genomic sequences of *C. faberi*, it is imperative to construct a high-quality yeast library for the further point-to-point verification and functional identification of target proteins. The longer the fragments, the more convenient it is to detect the exact functions of the integral target proteins.

A bHLH transcription factor, *CfbHLH36*, was screened by RNA-seq results and blasted with *AtJAM3* which is a negative regulator in MeJA signaling pathway in Arabidopsis [16]. Therefore, we would like to excavate the target interaction proteins and regulation mechanism of CfbHLH36 in *C. faberi*. A candidate protein homologous to a protein involved in pathogenesis was screened out by yeast two-hybrid assay with the constructed yeast library, suggesting that this library is suitable for searching unknown proteins with the bait proteins from *C. faberi*.

## Results

### Extraction of total RNA from different tissues and flowering stages of *C. faberi*

**Fig. 1:**
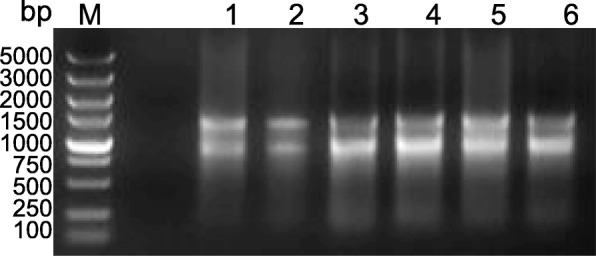
Total RNA extracted from flowers, leaves and roots of *C. faberi*. M: DL 5000 DNA Marker; 1: flowers in the bud stage; 2: flowers in the blooming stage; 3: flowers in the withered stage; 4 and 5: young leaves; 6: roots

Leaves, roots, flower buds, blooming flowers and withered flowers of *C. faberi* were collected for total RNA extraction. The quality of the total RNA samples is shown in Fig. 1, which obviously shows the bands corresponding to the intact 28S and 18S rRNA. The total RNAs had an A260/A280 ratio of ~ 2.0 and a concentration of 1.3~2.4 μg/μL, which fulfilled the requirements for library construction.

### The mRNA was purified and reverse-transcribed into double-stranded cDNA, which was then normalized to harvest confluent ds cDNA

**Fig. 2:**
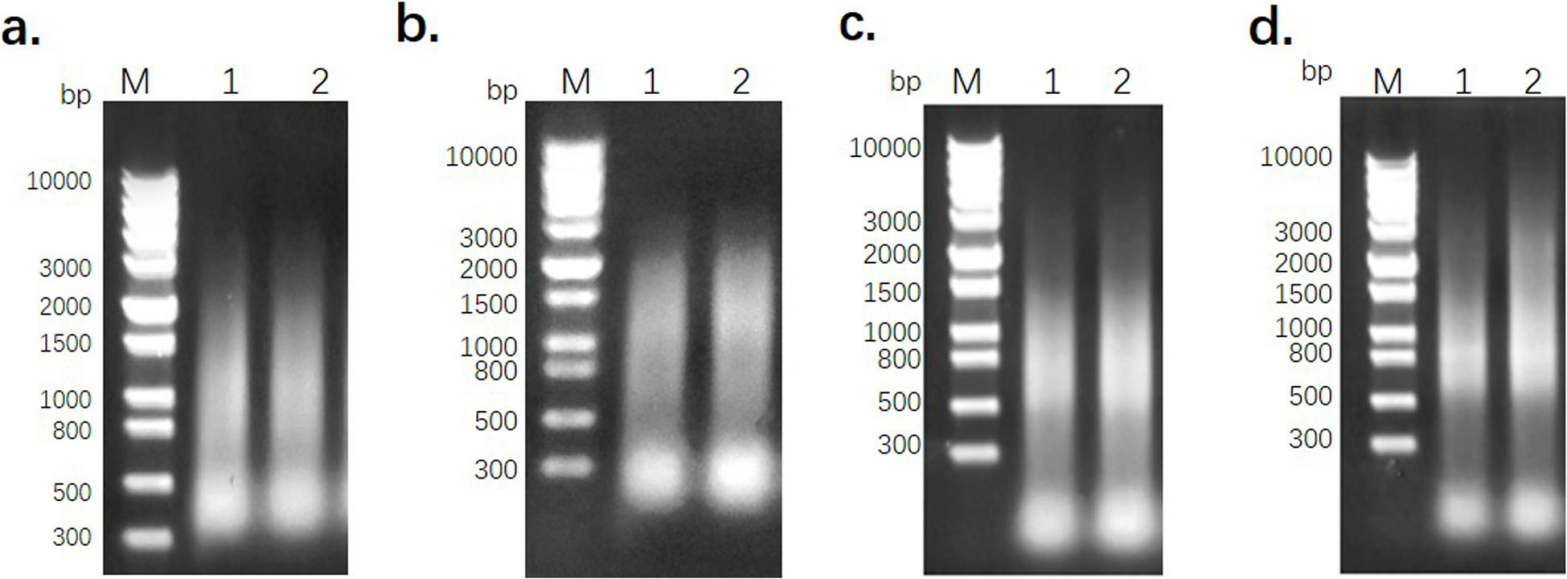
The synthesis, purification and normalization of cDNA. **a.** the purification of mRNA from total RNA; **b.** the double-strand cDNA synthesized by LD-PCR; **c.** the purification of cDNA using a CHROMA SPIN TE-400 column; **d.** the normalization of the ds cDNA. M: DL 10000 DNA Marker; 1: mRNA or cDNA from *C. faberi*; 2: positive control

As shown in Fig. 2a, the mRNA was purified from total RNA by adsorption to magnetic beads. The length of the extracted mRNAs ranged from 100 to 3000 bp. The mRNA was then reverse-transcribed into first-strand cDNA, which was used to synthesize the double-stranded cDNA by LD-PCR (Fig. 2b). The cDNA fragments ranged in size from 300 to 2000 bp. The ds cDNA was subsequently purified using a CHROMA SPIN TE-400 column as shown in Fig. 2c. The normalization showed that ds cDNAs were uniformly dispersed, without the disproportionate enrichment of specific fragments. Furthermore, the range of lengths was similar to that of the preceding non-normalized ds cDNA, except for the loss of short fragments smaller than 300 bp (Fig. 2d).

### The yeast one- and two-hybrid library was successfully constructed with a large library capacity and appropriate inserted fragments

**Fig. 3:**
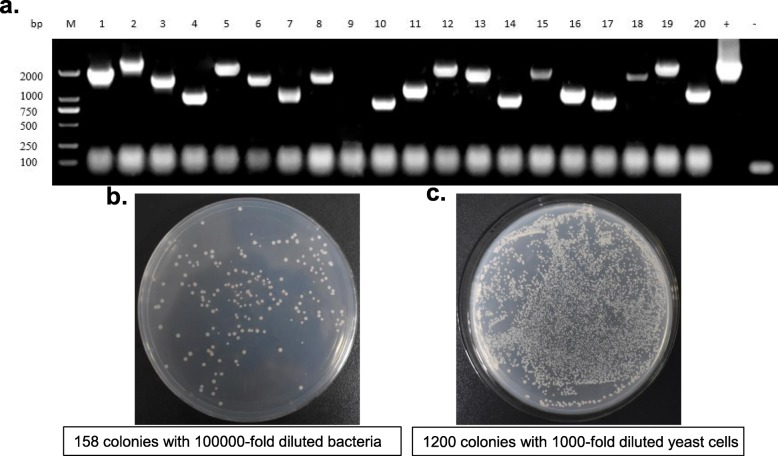
The quantification of the library by sequencing of positive colonies and plate counting. **a.** agarose gel electrophoresis of PCR products from randomly selected 20 colonies. M: DL 2000 DNA Marker; 1–20: PCR products of 20 colonies; +: pGADT7-T vector as positive control; −: ddH_2_O as negative control. **b.** plate counting of 100,000-fold diluted bacteria from the *E. coli* library. **c.** plate counting of 1000-fold diluted yeast cells from the yeast library

Homologous recombination was used to ligate the cDNAs into the pGADT7- *Sma*I vector to construct the three-frame cDNA library. After condensation and purification, the recombinant vectors were electroporated into competent cells of *E. coli* DH10B. The lengths of the inserted fragments ranged from 750 bp to 3000 bp, with a recombination efficiency of 95% (Fig. 3a). The transformed bacteria were diluted for plate counting and the result showed that the bacteria library was 1.58 × 10^9^ CFU/ mL (Fig. 3b). Eight of the 19 positive colonies were sequenced and blasted in NCBI database, and all of them were homologous to the corresponding proteins in other Orchidacea plants including *Phalaenopsis equestris* (XP_020598791.1, XP_020578536.1, XP_020573601.1, XP_020578326.1, XP_020579629.1), *Dendrobium catenatum* (XP_020688313.1) and *Cymbidium hybrid* cultivar (AAA19578.1) (Additional file 1). The library plasmid was then transformed into *S. cerevisiae* Y187 to obtain the yeast library, and the diluted yeast cells were grown on the YPDA plates. The counting result showed that the yeast library capacity was 1.2 × 10^8^ CFU/mL × 50 mL = 6 × 10^9^ CFU/μg of cDNA insert (Fig. 3c).

### One candidate gene related with pathogenesis was screened out from yeast library and might participate in MeJA-mediated biotic stress responses

**Fig. 4:**
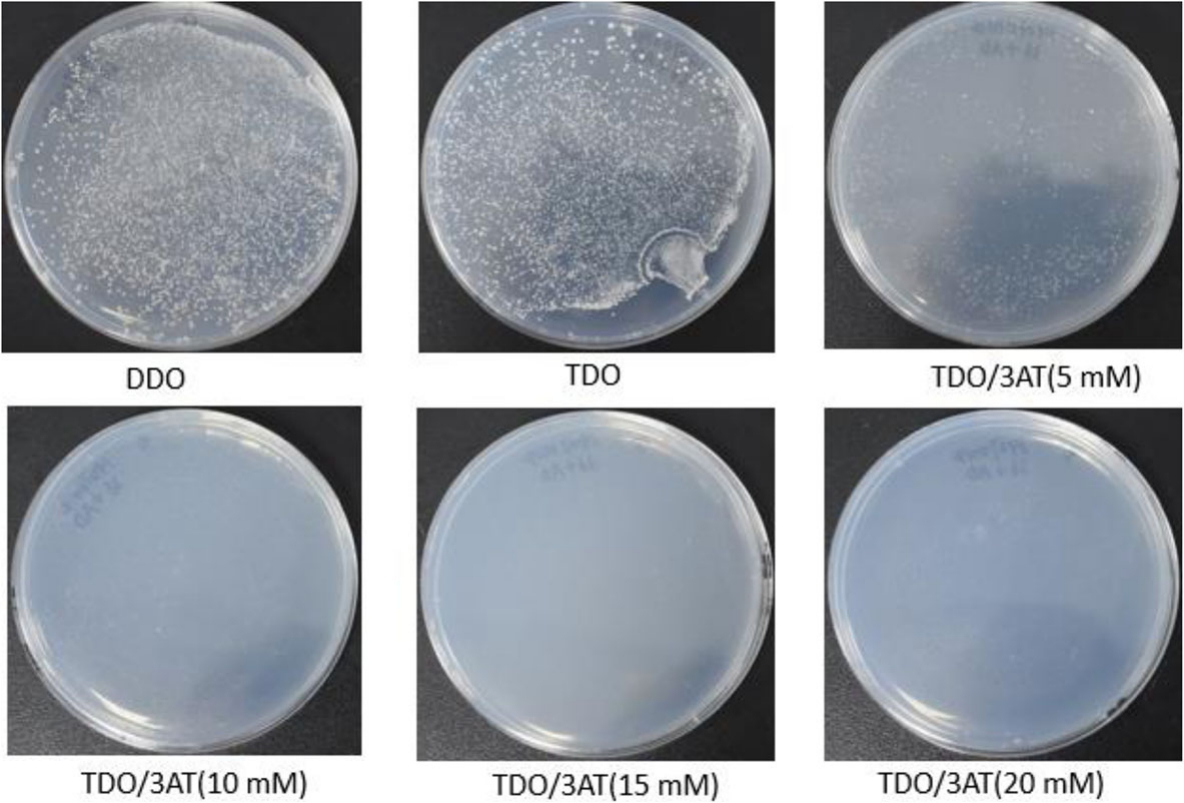
The toxicity and auto-activation detection of pGBKT7-CfbHLH36 bait vector. DDO: SD/−Leu/−Trp culture media; TDO: SD/−Leu/−Trp/−His culture media; TDO/3AT: SD/−Leu/−Trp/−His culture media supplemented with different concentrations of 3-Aminotriazole

**Fig. 5:**
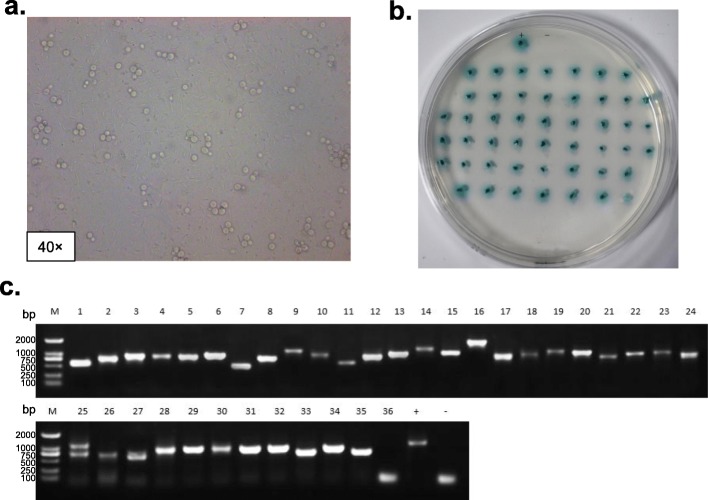
The screening of the constructed yeast library. **a.** the diploid yeast cells were grown into a typical clover-leaf shape stage; **b.** the blue positive colonies were grown in stringent QDO plates to increase the selection pressure. +: pGBKT7–53 and pGADT7-T co-transformed as positive control; −: pGBKT7-Lam and pGADT7-T co-transformed as negative control; **c.** agarose gel electrophoresis of PCR products from selected positive colonies. +: pGADT7-T vector as positive control; −: ddH_2_O as negative control

CfbHLH36 transcription factor related with the MeJA signaling pathway in *C. faberi* was selected to construct bait vector for library screening. The recombinant plasmid of pGBKT7-CfbHLH36 could be grown on SD/−Leu/−Trp plates with prey vector of pGADT7, suggesting that the recombinant plasmid could be successfully transformed into the host cells with no toxicity. But its growth on SD/−Leu/−Trp/−His plates showed that the recombinant plasmid could autoactivate the expression of *His* reporter gene, which could be inhibited by 10 mM 3′ AT (Fig. 4). The subsequent screening plates would observe this growth condition. As shown in Fig. 5a, as the bait vector and the Y187 yeast library grown into a typical clover-leaf shape, the yeast zygotes were cultured onto the SD/−Leu/−Trp/−His/X-α-Gal plates with 10 mM 3’AT for selection. Then the positive colonies were grown in blue for the *LacZ* reporter gene could catalyze the X-α-Gal supplemented in the media.

About 50 blue colonies were grown in stringent QDO plates (Fig. 5b), of which 36 colonies were identified by PCR and sequencing. As shown in Fig. 5c, the inserted fragments were ranged from 500~2000 bp in length via electrophoresis. The positive colonies were sequenced and blasted in NCBI database. Among them, one of the proteins homologous to a pathogenesis-related protein derived from *Phalaenopsis equestris* (XP_020589637.1) was screened out (Additional file 2).

## Discussion

In this study, we constructed a high-quality cDNA library that can be used for both yeast one- and two-hybrid assays, which provides a solid foundation for functional identification of unknown proteins in *C. faberi*. The final product of gene expression often interacts with other proteins or DNAs to form a complex, resulting complicated regulation of gene expression that enables organisms to survive in different environments. The high-throughput screening of interactions between target proteins and a bait vector enables the fast and efficient identification of links in the complicated and elegant regulation networks of gene expression found in higher organisms.

In order to improve the efficiency of transferring cDNA fragments into multiple destination vectors, several yeast one- and two-hybrid systems have been modified to use Gateway technology, In-Fusion technology and so on [17–19]. Moreover, in recent studies two cDNA libraries were respectively used as bait library and prey library, followed by mating and screening, enabling the screening of multiple libraries in one pool. Examples include BFG-Y2H [20], CrY2H [21], and RLL-Y2H [22]. The continuous improvement of the construction methods makes the information included in the library more comprehensive and more integral.

The crucial elements for the construction of a high-quality yeast library include the purity, integrity and concentration of mRNA, the ligation efficiency, and the transformation efficiency. The standards used to judge the quality of a yeast library are mainly based on the recombination efficiency, the lengths of inserted fragments, and the library capacity. In general, a cDNA library that includes the integral expression information must contain at least 1 × 10^6^ CFU [23]. In this study, the three indexes of the yeast library of *C. faberi* were 95%, 750~3000 bp and 6 × 10^9^ CFU/μg of cDNA insert, respectively, which fulfilled the requirements of the subsequent screening. The corresponded three indexes of a recently published yeast library of *Salvia miltiorrhiza* were 100%, 500~2000 bp and 1.45 × 10^6^ CFU/mL, respectively [24]. The yeast two-hybrid library from tobacco leaves infected by *Lasiodiplodia theobromae* had a size of 1.2 × 10^8^ CFU/mL, with inserted fragments of 350~2000 bp [25].

In order to avoid creating a limited cDNA library due to restricted spatiotemporal expression, in this study the total RNA was extracted from different tissues and different flowering stages, which could enlarge the screening scope, especially for crucial genes related to flower development. Correspondingly, the quantity of the primary RNA used for reverse transcription should be sufficiently large. The cDNA fragments were randomly inserted into the library vector pGADT7, which probably leads to frame-shifts of the expression of the cDNA. In this study three different library vectors, pGADT7-*Sma*I-1, pGADT7-*Sma*I-2 and pGADT7-*Sma*I-3, were respectively ligated with the cDNA, which could increase the efficiency of screening out target proteins with the correct encoding frame. This step also required a high quantity and quality of the cDNA. Therefore, it is necessary to prepare a good cDNA library in advance.

**Fig. 6:**
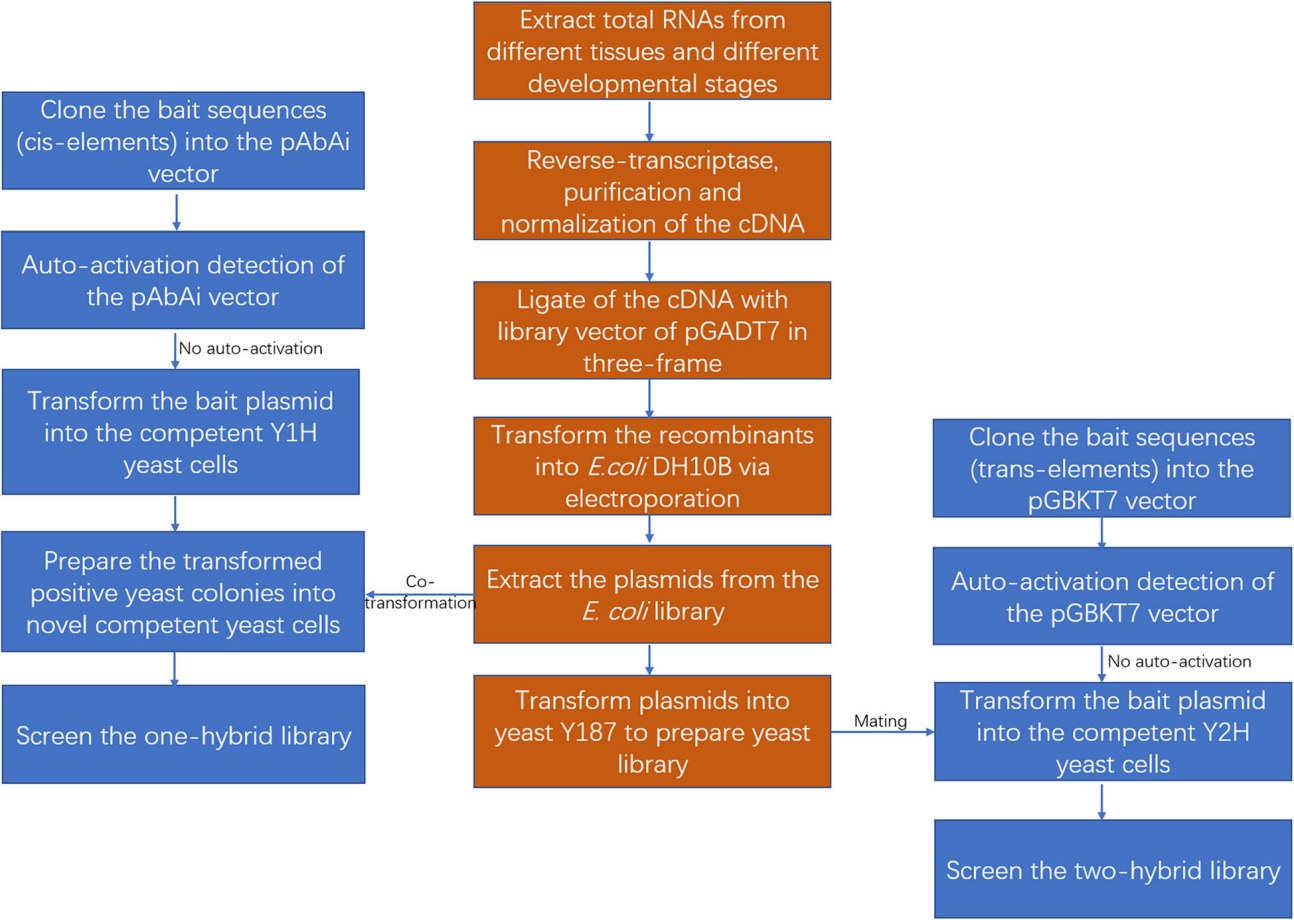
Flowchart of the construction and screening of the universal Y1H and Y2H yeast libraries

Since a yeast two-hybrid system comprises an expressed cDNA library, it is also compatible with yeast one-hybrid screening. In this study, the cDNA library could be used to construct both yeast one- and two-hybrid systems. As shown in Fig. 6, the universal yeast library in this study was constructed based on a large, high-quality cDNA library derived from different tissues and developmental stages of *C. faberi*. The library plasmids could be electroporated into the Y1H yeast competent cells generated from a colony containing a cis-element-based bait plasmid. The yeast one-hybrid screening was based on the activation of the aureobasidin resistance gene in the pAbAi vector, and positive colonies were selected on plates with a gradient of aureobasidin concentrations [26]. For the yeast two-hybrid assay, the bait vector was electroporated into a sexually compatible haploid yeast strain (Y2H Gold with the “a” mating type), and the yeast library was constructed in the strain Y187 (α mating type). Consequently, mating could be used to combine the bait and prey constructs in the same diploid yeast cells [9]. The diploid yeast cells displayed a typical clover-leaf shape under the microscope. In this case, the target proteins could be screened out from one library, and sometimes the screening results of Y1H and Y2H could be mutually confirmed for the same metabolic pathway.

Methyl jasmonate (MeJA) participates in many processes of plant development, biotic and abiotic responses [11, 27, 28]. In this study, a target protein, homologous to a pathogenesis-related protein in *Phalaenopsis equestris* (XP_020589637.1) was screened out based on the CfbHLH36 transcription factor as the bait vector. CfbHLH36 transcription factor is involved in the MeJA signaling pathway, for it is homologous to AtJAM3 in Arabidopsis and its expression pattern is negatively correlated with the contents of MeJA in different flower development stages of *C. faberi* (unpublished data). As MeJA plays a crucial role in the plant responses to external stimuli [29], we expected that CfbHLH36 transcription factor may participate in the MeJA-mediated interaction between plants and microbes in *C. faberi*. But the exact function mechanism remained to be explored in the further research.

## Conclusion

A universal three-frame yeast library of *C. faberi* was successfully constructed, which provides a useful tool for the research on functional genomics of *C. faberi*, especially targeting transcription factors and related proteins in MeJA signaling pathway and flower development.

## Methods

### Plant materials

Plantlets of *C. faberi* were collected from one wild population in Dangyang, Hubei province (30°55′25″N, 111°51′24″E), China, which was identified by taxonomist professor Yanqin Xu. They were then transplanted and divided propagated in the greenhouse of Wuhan University of Bioengineering. Herbarium specimens of *C. faberi* were deposited in the herbarium of Jiangxi University of Traditional Chinese Medicine (Y. Zhou & Y. Q Xu 20,120,301).

### Extraction of total RNA from different tissues of *C. faberi* and first-strand cDNA synthesis

Fresh samples of *C. faberi*, including young leaves, root, and flowers in different stages (flower bud stage, blooming stage and withered stage) were collected and ground into powder after freezing with liquid nitrogen. The total RNA was extracted according to the Trizol Extraction Protocol (Takara, Dalian, China). The extracted total RNA (~ 200 μg) was diluted in 100 μL DEPC ddH_2_O, incubated at 65 °C for 2 min and stored on ice. Total RNAs from different tissues and different stages were mixed together for the subsequent purification. The mRNA was purified via the poly A tail using the NucleoTrap mRNA kit (Clontech, CA, USA) according to the manufacturer’s instructions. After washing with 200 μL of washing buffer twice, 10~20 μL 10 mM Tris-HCl (pH 7.5) was added to elute the mRNA at 80 °C for 2 min. The first-strand cDNA was synthesized using the SMART III reverse-transcriptase kit (Takara, Dalian, China). The reverse-transcriptase reaction was terminated at 75 °C for 10 min, after which 1 μL RNase H (2 Units) was added and incubated at 37 °C for 20 min to digest the redundant mRNA.

### The synthesis of double-strand cDNA using LD-PCR, purification and normalization

The double-strand cDNA was synthesized using long-distance (LD)-PCR containing 2 μL of first-strand cDNA, 10 μL 10 × Advantage 2 PCR buffer, 2 μL 50 × dNTP Mix, 2 μL 5′ PCR primer, 2 μL 3′ PCR primer, 10 μL melting solution, 2 μL 50 × Advantage 2 polymerase mix (Clontech, CA, USA), and ddH_2_O up to a volume of 100 μL. The PCR temperature program encompassed an initial denaturation step at 95 °C for 30 s, followed by 20 cycles of 95 °C, 10s, 65 °C, 6 min (each cycle increased by 5 s), and a final elongation step at 68 °C for 6 min. Following detection via agarose gel electrophoresis, the rest of the double-strand cDNA was added into the CHROMA SPIN TE-400 column (Takara, Dalian, China) for purification. The purified cDNA was precipitated with 1/10 volume of 3 M NaAc and 2.5 volumes of ethanol, and then stored at − 20 °C for 1 h. The precipitate was resuspended in 20 μL ddH_2_O and examined via agarose electrophoresis. The double-strand cDNA was normalized using the Trimmer-Direct cDNA normalization kit (Evrogen, Moscow, Russia). The 4 μL 4 × hybridizaiton buffer was added into the cDNA and incubated at 98 °C for 2 min and then at 68 °C for 5 h. Then, 4 μL of 4 × DSN buffer and 0.2 μL duplex-specific nuclease (DSN) (1 U/μL) were added into the PCR tube and incubated at 68 °C for 3 min. The normalized product was extracted with phenol and chloroform once and finally diluted in 20 μL DEPC ddH_2_O.

### Ligation of double-stranded cDNA with library vectors to construct the three-frame library

The normalized cDNA was divided into triplicates and ligated with linearized pGADT7-*Sma*I- 1, pGADT7-*Sma*I- 2, and pGADT7-*Sma*I- 3 vectors, respectively, by homologous recombination. The ligation system contained 7 μL cDNA, 3 μL library vector DNA, 5 μL In-Fusion recombinase (Clontech, CA, USA), and ddH_2_O up to 20 μL, and was incubated at 50 °C for 1 h. The ligation reaction was terminated by adding 2 μL proteinase K (Sigma-Aldrich, USA), followed by the addition of 1 μL 20 μg/μL glycogen (Sigma-Aldrich), 50 μL 7.5 M NH_4_Ac, and 375 μL ethanol, and stored at − 80 °C for at least 1 h. The ligation product was condensed and resuspended in 10 μL DEPC ddH_2_O on ice.

### Electroporation of the recombinant libraries into *E. coli* DH10B and *S. cerevisiae* Y187

The 1 mm electroporation cuvettes were precooled on ice for 30 min, after which 2.5 μL recombinant vector DNA and 50 μL competent cells of *E. coli* DH10B were mixed in the cuvettes and electroporated at 2000 V for 5 ms. After that, 1 mL of LB medium was rapidly added into the cuvette. The transformed bacteria were gently mixed with liquid LB and the medium volume increased to 5 mL, followed by incubation at 37 °C for 1 h. The cultured bacteria were diluted 10-, 100-, 1000-, 10,000- and 100,000-fold and spread on LB agar plates with ampicillin (Sigma-Aldrich). The rest of the bacteria were stored at − 80 °C. The plasmid library was extracted using a plasmid-purification kit (Qiagen, Valencia, CA) and then used to transform *S. cerevisiae* Y187 to construct the yeast library following the same electroporation method described, but with YPDA instead of LB.

### Determination of the library capacity and the average length of the inserted fragments

A sample comprising 10 μL of 100,000-fold diluted bacteria was used for plate counting. The library capacity was calculated according to the formula CFU/mL = colonies on the plate/ 10 μL × 100,000 × 1 × 10^3^. The total CFU of the library = CFU/mL × total volume of library (50 mL). A total of 20 randomly selected colonies were picked to amplify the inserted fragments of the library. The amplification primers were designed based on the pGADT7-Rec vector (T7-F: 5′-GGAGTACCCATACGACGTACC-3′ and T7-R: 5′-TATCTACGATTCATCTGCAGC-3′), compared with pGADT7-T vector as positive control and ddH_2_O as negative control. The lengths of the inserted fragments were analyzed via agarose gel electrophoresis. Eight out of 19 positive colonies were sequenced at Sangon Biotech (Shanghai, China). The sequencing results were blasted in the National Center for Biotechnology Information (NCBI) database to identify their resources and closely related functions in other species. The similar calculation method was applied to count library capacity of yeast except that the yeast cells were from 10 μL liquid of 1000-fold diluted.

### The auto-activation and toxicity detection of bait vectors

In order to identify the methyl jasmonate signaling pathway in *C. faberi*, a candidate gene, *CfbHLH36*, selected based on RNA-seq results were cloned and inserted into bait vector of pGBKT7 with *Nde*I and *Sal*I restriction sites. The ligation products of pGBKT7-CfbHLH36 were transformed into *E. coli* DH5α and the positive recombinant colonies were selected to extract plasmids. Following the identification and sequencing of the recombinant plasmids, about 100 ng recombinant plasmid of pGBKT7-CfbHLH36 and 100 ng prey vector plasmid of pGADT7 were co-transformed into competent cells of Y2H Gold yeast to detect the autoactivation and toxicity. The transformed competent cells were then cultured into media of SD/-Leu/-Trp (DDO), SD/-Leu/-Trp/-His (TDO) and SD/-Leu/-Trp/-His plates with 5 mM, 10 mM, 15 mM and 20 mM 3′ AT, respectively at 30 °C for 3-4 days. The diameters and colors of colonies were observed and recorded.

### The screening and identification of positive interactors in Y2H library

The fresh colony of bait vector pGBKT7-CfbHLH36 was selected and cultured in 50 mL SD/-Trp media at 30 °C, 250 rpm until the OD_600_ reach to 0.8. Then the Y2H Gold yeast cells were collected and resuspended in 5 mL SD/-Trp media, which was prepared for the mating with 1 mL cDNA library in 45 mL 2 × YPDA media at 30 °C, 50 rpm for 20 h. The growth of the zygotes was observed and recorded with inverted microscope (XDS-1A, Shanghai Precision Instrument Co., Shanghai, China). Compared with the growth condition of positive and negative controls, the yeast zygotes were collected and cultured on 50 SD/-Leu/-Trp/-His/X-α-Gal plates (TDO/X) with 10 mM 3’AT at 30 °C for 3-5 d. The candidate blue yeast cells were selected and transferred into stringent SD/-Leu/-Trp/-His/-Ade/X-α-Gal plates (QDO/X) with 10 mM 3’AT at 30 °C for 3 d to increase the selection pressure, compared with pGBKT7-53 and pGADT7-T co-transformed as positive control, pGBKT7-Lam and pGADT7-T co-transformed as negative control. Finally, about 50 blue colonies were randomly selected, cultured in SD/-Leu/-Trp/-His/-Ade liquid media (QDO) and identified with PCR and sequencing, compared with pGADT7-T vector as positive control. All of the reagents used in yeast culture and library screening were bought from Clontech company (CA, USA). All of the restriction digestion enzymes were bought from Takara Company (Dalian, China). The PCR products were sequenced at Sangon Biotech Company (Shanghai, China).

## Supplementary information

**Additional file 1.** The sequencing and blast results of the randomly selected eight colonies in library construction.

**Additional file 2.** The sequencing and blast results of the 20 selected positive colonies in library screening.

## Data Availability

The cDNA library of *Cymbidium faberi* in this study can be available to researchers upon reasonable request to the corresponding author.

## Abbreviations

3’AT: 3-Aminotriazole
CFU: Colony-forming units
DDO: SD/−Leu/−Trp
DEPC: Diethyl pyrocarbonate
DSN: Duplex-specific nuclease
LD-PCR: Long distance-PCR
QDO: SD/−Leu/−Trp/−His/−Ade
TDO: SD/−Leu/−Trp/−His
TF: Transcription factor
Y1H: Yeast one-hybrid
Y2H: Yeast two-hybrid
