# Supplementary material for: Construction and characterization of a high-quality cDNA library of Cymbidium faberi suitable for yeast one- and two-hybrid assays
Source: BMC Biotechnol. 2020 Jan 16;20:4. doi: 10.1186/s12896-020-0599-2 (PMC6966867; doi:10.1186/s12896-020-0599-2)

1：

>0605-N46(19030028-1)T7

GGGCCGGGAGTCCATACGACGTACCAGATTACGCTCACAAGTTTGTACAAAAAACCAAGCAGTGGTATCAACGCAGAGTGGCCATTATGGCCGGGGCTCCTCTTCCAAACTCGTCCTCCGGCGAGCATCTAACTTGGAGCTTGTCGTTTTCTTGCGTCTAGCAATCTCGGTTTTGGTTTATGAGCTCGAAGGAGAAACCGACTCTCGGTGGCACGCGGATTAAGCCCCGCAAACGGAATATCACCGCTCCTTTGGACCCTGCAGCATTTGCGGATGCAGTGGTCCAGATTTATTTGGATAATGCTGGTGATCTGGAACTTGTTGCCAAAAGCATCGAATCTTCAGATCTTAATTTCTCCAGATACGGTGAAACCTTTTTTGAGGTCATCTTTACTGGAGGCCGAACTCAACCTGGCACAATAAAGTCTGATGAAGGAGATAGGCATTCTTATTCTGTTATAGATTGTGAGGCAACACGTGAAGCTATATTACCTTCTGTTCTCTATATACAGAAAATCTTGCGTCGTCGACCTTTTCTAATTAAAAACCTAGAAAATGTCATGCGAAGATTTTTACAGTGTTTGGAACTATTTGAGGACAATGAAAGGAAGAAGCTTGCTATTTTCACTGCCCTTACATTTTCTCAGAAGCTATCTGGTCTTCCTCCAGAGACTGTTTTCCAGCCATTGCTTAAGGATAATCTTGTTGGCAAGGGGCTAGTTCTTTCATTCATTACTGACTTCTTTAAGGAGTATCTGAAGGACAGTAATATAGATGATCTTATTGCACTCTTGAAGAAAGGGAAAGATGGAGGATAATATGCTTGAATTTTTCCCAACTGCAAAACGCACTGCTGAAGGTTTCTCTGAACATTTCACAAAAGAAGGATTGGCTTCTCTTGTTGAATACAATCAAAAGAAAATTTTTGATGTCAGCTAAAGAGATGAAGTCAGCATTGAAAACTCAATTGAGAAGAGATTGATATTTTCTGAAGTTATAGAGACTATGAGCACAGTACGATCTATATGCTGAATATGAGTGTACCGCTTGCTGTGGATGTGCTAATGGATGCAGTTCAGTGTTCGGGAAAGAAACAG


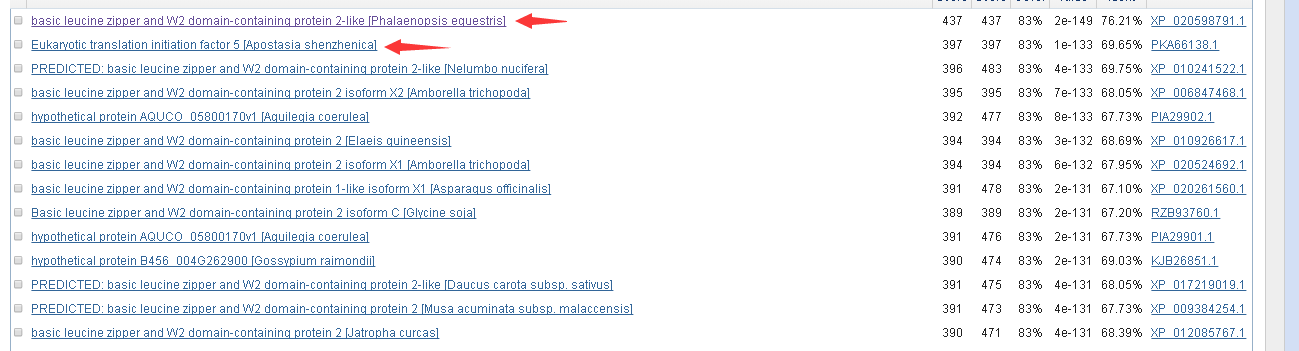


2：

>0605-N47(19030028-2)T7

TCCGGGCCCCTCGTATGTTTATTGCGCTCACGAGTTTGTACAAAAAACCAAGCAGTGGTATCAACGCAGAGTGGCCATTATGGCCGGGGGCTCTTCGCTTCTTCTAACAGTGGTTCCGGCGGCACAGGCAGCGGCGGGGGGAATGGGTGAGAGCTTGTTCTCGTCGGACCCGACATCGTTGAGGATGGATAGGAGATCGCCGGAGGCGGTTAGGTTGCGAGAGGCGGTCTCTAAGAAGCTGTTGGAGTTCCTCGGAAATTACTCCGACGACGTCCTAGCGGAGTACATTGTGGTGCTTGTTTGTAATGGCAAGCATCAGAATCAAGCACGCGATGACCTACAAGCATTTTTAGGGGAGGAGAGCGGCACATTTGTAGCTTGGCTTTGGGATTATCTTTCCAAAGAATTCATTTCAAAGACGACTCCTGCCTTCTCGGATCTAGAAGCCAAAGTAACAACTAATCATAGTAACAGTTTGGACAGGGAGTATAAACATAGCATTCTGGCAGACCATGCTGCTCAATCAGTTGGGTCGCCAAAGACTAAGAATGGATCATTGGAACTGCCATCCTGTAGTACAACTTCAGGCTACTCGAAACCTCCCCAAGTGACAGAGAGACTCCAGCTTCATCCCCCAAATTTAGATGAACGATCAACTGCAGTAAATTTGGAGAGGCTGAATGCACAAGAGATTGCTCTTCGGAAGAGTTCAGGTTCAAAGAGCGAAGAGCATCAATTAGGGCCTTCGTCCGATAGATATTTTACTGAAACAAGGCTGCCTTCAACTGCTATCAATGATTTATCACATCAAACAGCTCGTTTACGTGGCAATGTTTGGGATAGATTAGGAAGGCCTCATGTAGAAGATAAAATACCAGTTAAAGAAGAGCAATCCGATCATCTTGATTTCATTGATGGTGGAGAGCTAGACCATCGAGAAGAACTTCAAATCTCAGAACTGCAGATAAAGTGATTGATGCAAGTACATTTAAAACGAAGAGAGATATGGGTGAGCTAACACCAACAATAGCTCATCCTGCAAAGAGAATCTTCAAGACAAGGAACATTCTCCAGCATCC


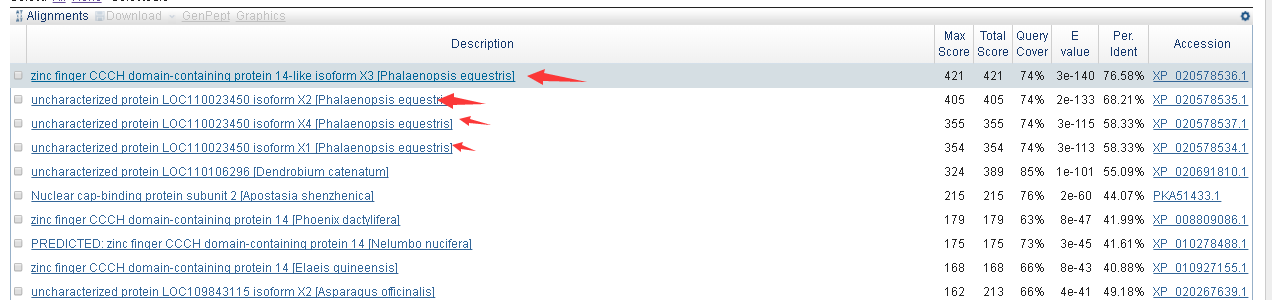


3：

>0605-N48(19030028-3)T7

CCATTACGGAACGTACCAGATTACGCTACAAGTTTGTACAAAAAACCAAGCAGTGGTATCAACGCAGAGTGGCCATTATGGCCGGGGGGCGTCGCAACGCTGTAGTATCGGATGGTTCTAACAGACGGCCATGGAGTCGAACCGGCCGCCCGAAATGCTACAGAAGAGCCAGCGCTTCGCCAGCGGACAGATGCCGCCGAATCCTCGAAGCTCCGGCCATCGGCGGGCCCACTCGGGGAACTTCTTCCACCTCCCCGACGAGTTCCTCTTCGACTCTGAACCCGATTTTAACTTCTCCGATGTCGAGTTACCCTCTGTTTCAGATGATAACATCTCCGGCAGCAACTCCATCGCCGATGCAGCCACTTCCGCCGATTCCGGTGGCACATCAAAGTTGCTGGCGGTGCATCCGGTGTCGAGGCCTTCCGGAGGTGGAGTTCACCTGAGGAGTCTATCGTTGGACACGGCGTTCTTCGATGGGCTGGGGTTCCAGGGACCCGGCGCCACCAGTGTTTCGGCACCGGAGAAGAAGCCGCAGCATCGGCGCAGCATCTCTATGGACGGAGTGACTTCCCCTTTCGAAGGGGAATCGATGCCACCGTCTTCGAATTATTCAAAGACGGCGGTGGCGATTGATAAGCTTGCGGAGTTGGCTCTAATCGATCCGAAGAGAGCCAAAAGAATTCTTGCGAACAGGCAGTCTGCTGCTCGATCCAAAGAGAGAAAAATATGCCACACAAATGAACTTGAGCGGAAGGTGCAGACACTTCAGACAGAGGCAACAACTCTCTTGGCTCAGCTTACCTTGCTTCAGAGGAATTCTACGGACTTAAACACTGAGAATAGAGAACTAAAACTGCGGTTACAGTCTATGGAACAACAAGCTCATCTCAGGGTGCTTTGAACGAGCGTTGAGAAGAAGAAGTTCACGGCTCAGATGGCAACTTTACAGCATCCAAGCATCAATGGCGGCACCTTCAACCTGTGTTCAGCACTGTCCTCTTCTTATTTCTCTCATCCACAACAGTTCTAGTCAGCAGACTCAGCAGATGCATTCAGCTCCACTTAACGGTCATCT


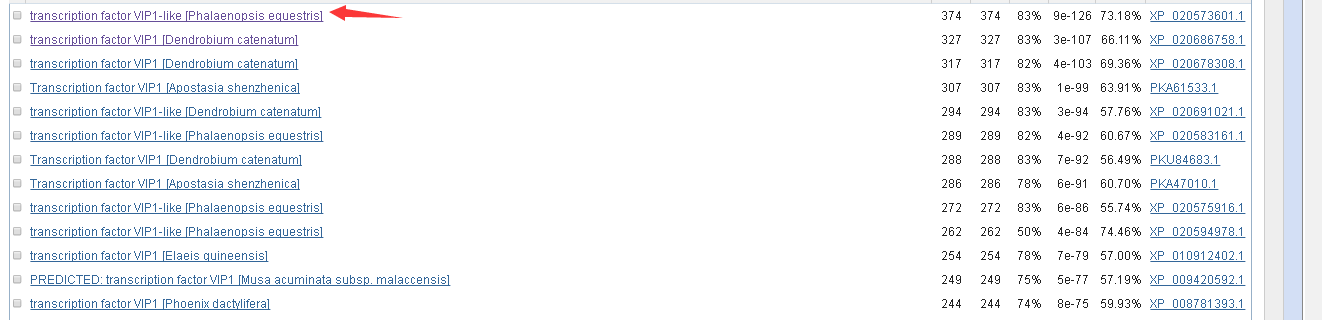


4：

>0605-N49(19030028-4)T7

TCCATTACGGACGTACCAGATTACGCTACAAGTTTGTACAAAAAACCAAGCAGTGGTATCAACGCAGAGTGGCCATTATGGCCGGGGAGACAAACCAACCCCCTAATTAATTAACCAGCCATGGGTATCTTCTCCATTATCAGGACCCTTCTCCTCTGCGCCGCATCCCTCACAATCCTTCTTGCCAACCCATCGTCCGGCCAGTGCAACAACCACTTGCTCTCCGGTGAGCGCCTTAGCCCAGGACAATCCCTCACAAGTGGCAACATGGAGTTCATCATGCAATACGACTGCAATCTCGTCCTCTACGATAACGGGAAACCGATCTGGGCTTCGGGCACCTATGGTAAAGGTTCCAGCTGCTACGTCGCCATGCAGACCGACGGCAACCTCGTCGTCTACGACAATAGAAATAATCCCTTATGGGCGAGCAACACTGGTGGGGAAAATGGAAACTATATCCTTATACTGCAAAAAGATCGCAATCTTGTTATATACAGCCACCCCATTTGGGCTACGGGGACTAATTACGCTGGTTCGGTCGCTGTCGTCGTGGCCGCTGCGCGTAATGGGACGGTGGGGATTTCGGGGGCGGAGCAGAACAAGGTGAGTGAAATAAGGAAGATTCTGAAGATGAGTGTTGAATAGGCGAGGCGTGTGCGTATGCGCGAGGGTGAATAAGATAAGCGTGTATATGATAATATGCATGCATGCTGCGTGTATGGGTTTATGTTATCTGAAGTACGTCTGTAATTTCCTTGTTGCGTACGTGCGTTCTTATCGTTGAAATAAAATGGATGGCTGTGGTCGGCTTGAAAGCTGACAAAAAAAAAATAAAAAAAAAAAAACCTTGTCGCGCGCCCTCGGGCCTCTAAAGGTTTTGTGGACAAAGGGGGGGGGCTGGAAATGATTCGAAAATCCTGGAAAACCCCCGCAGTTCCCTTCTATTGGGCATCGGGGCCCTTATTTTTTGTAA


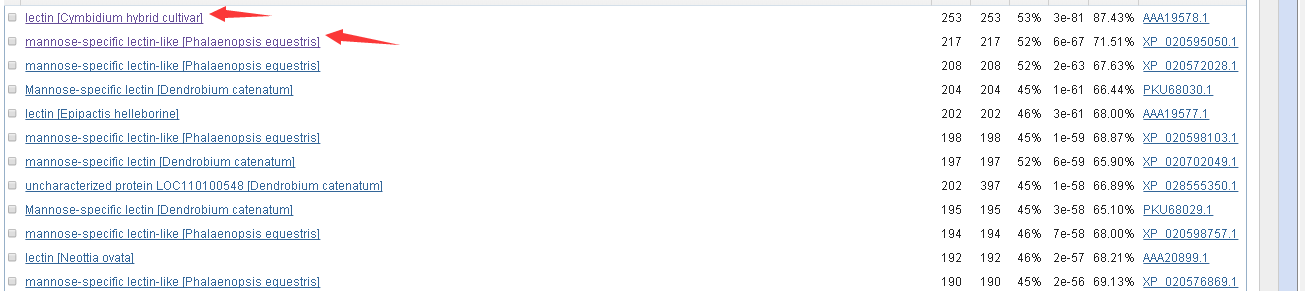


5：

>0605-N50(19030028-5)T7

GGGCCGGGGGGTCCCTACGACGTACCAGATTACGCTACAAGTTTGTACAAAAAACCAAGCAGTGGTATCAACGCAGAGTGGCCATTATGGCCGGGGATCGTTGAGATAAGTTCTCATTTAGAGAAGAATGGAGAAACACATTGTTCTTGCTCTCACCATTGTTCTTTGCCACTTTGCTCTCTCTTCCGCCATTGATGGAACTGCAACTTACTACACTCCACCTTATACACCTTCTTCATGTTACGGGTATCAAGATATGGGTACGATGATTGCTGCGGCGAGTGATGCTATTTGGGATAATCGTGCGGCGTGCGGACGGAATTATAGGGTCACGTGTACCGGTCCGACCAATCAAGGTGTGCCTCACCCATGTACAGGAGCAAGTGTTGTCGTCAAAATTGTTGATTATTGCCCTGCTGGTTGTCGAGGCACTATCGATCTCTCCCAAGAAGCTTTTGCAGCTATAGCGGATTTAAACGCTGGCAAAATCGAAATTGATTATACTCAGGTGTAGAGATGACTGGTGGCTGAGGAAAATCGGGCTCTAATATACTTCAAATCTATCAAAATAAGTGCGGAAGTATAGAAGGACTATATCTTTTTCTTAATACATAGAAATAAAGAATCTTCAAATAATAATGGATTATGCGACCAGCATAATAATATAGTTTGTATAGAACTTTGATTGTGCTTCCACTGAGCACAAATGTCTCTCCATCAATAAATATGCTTTTTCATTTTTCGCCCAAAAAAAAAAATAAAAAAAAAAAACCTTGGCGGCGCCCCCTCGGGCTTCAAGAGGTTCTTGGACCAAAGGGGGGGGCTCGCAATAGATTCGAAAAATCTGAAAAACCCGCAGGTTCCTTTCAATTTGTGCATCGGGGCCACACATTAAAA


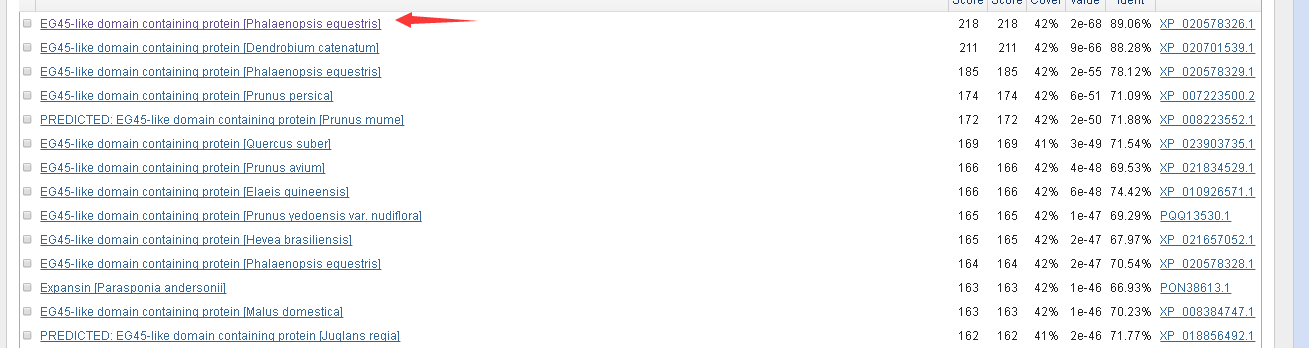


6：

>0605-N53(19030028-7-2)T7

ATTTTGCGACGTACCAGATTACGCTACAAGTTTGTACAAAAAACCAAGCAGTGGTATCAACGCAGAGTGGCCATTATGGCCGGGGGAACCAAGTAAATCAATACCCAAACTCCCAACTAATCAACACAGCCATGGCCTCCTCCACCTGCTCAGCAGCAGCCCTCCTCCTCCTCTCCGCGGCAGCGCTGTTGAGCCTCCTAACCACTCCCGTCTCCGCCGACGACCGCCTGAACGCAGGCCAGTCCCTTGAGGGGGGACAATCCCTCGCACAAGGCCCCTACTTGTTCACCATGCAGCAGGATTGCAACCTCGTCCTCTATGACAACAACGGGGCCATCTGGGCGACGGGGACCAACGGAAAAGCCTCCGGCTGCGTCGTCACGATGCAGACCGACGGCAACCTCGTCATTTATAGTGGCAGCAGTGTTATCTGGGCAAGCAACACCAACCGCCAGAATGATAACTACTATCTCATCCTCCGGAGAGATCGCAACGTCGTCATCTACGACAGCTCCAACAACGCTATTTGGGCGACTGGCACCAACGTCGGCAATGCTGCTGTTGTCGTCATCCCTCACAGCAACGGCACGGCGGCTGCGTCTGGCGCTGCGCAGAACAAGCTCAAGGAACTGTATCCATGAAGGCTAATATCACAGAGAGGAAGTATTCTTAATATATTTTGAATAAATTGGCGATGACAACTGTGGTGCTATTGCCTAACGCAAGCTAATTTTCCTTTTTATAACTGTGTTGTCGTAGTATGAATGATCGTATGAAATAAAGTTGCTTGTTTAAATTACC


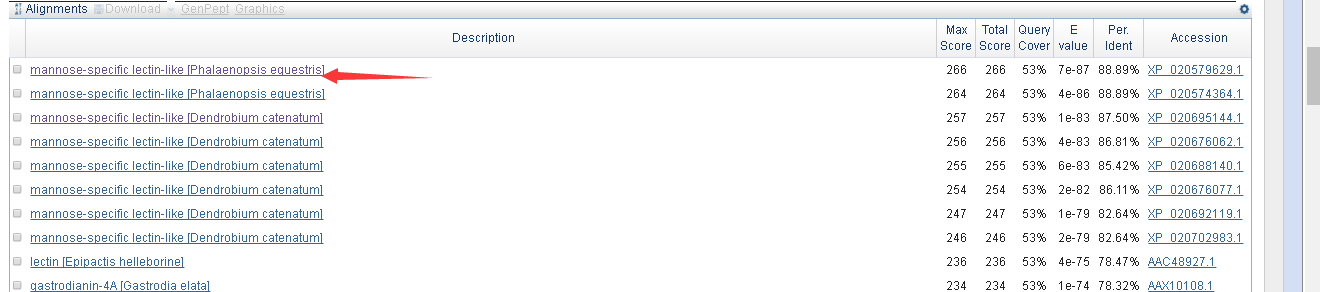


7：

>0605-N54(19030028-8)T7

CCCGTACGACGTACCAGATTACGCTACAAGTTTGTACAAAAAACCAAGCAGTGGTATCAACGCAGAGTGGCCATTATGGCCGGGGGATCGTTGAGATAAGTTGTTAGTATTCTCTCTCATTTAAAGAAGAATGGAGAAACACTTTGTTCTTGCTCTCACCATTGTTCTTTGCCACTTTGCTCTCTCTTCCGCCATTGATGGAACTGCAACTTACTACACTCCACCTTATACACCATCTTCATGTTACGGGTATCAAGATATGGGTACGTTGATTGCTGCGGCGAGCGATGCTATTTGGGATAATCGTGCGGCGTGCGGACGGAATTATAGAGTCACGTGTACCGGACCGACCAATCAAGGTGTGCCTCACCCATGTACAGGAGCAAGTGTTGTCGTCAAAATTGTTGATTATTGCCCTGCTGGTTGTGGAGGCACTATAGATCTCTCCCAAGAAGCTTTTGCAGCTATAGCGGATTTAAACGCTGGCAAAATCGAAATTGATTATACTCAGGAATAGAGGTGACCGGTGGCTGTGGAAAATCGGGCTCTAATATACTTCAAATTTATCAAAATAAGTGCGGAAGTATAGAAGGACTATATCTGTTTCTTAATACATATAAATAAAGAATCTTCAAATAATAATGGATTATGCGACCAGCATAATAATATAGTTTGTATGGAACTTTGATTGTGCTTCCACTGAGCACAAATGTCTCTCCATCAATAAATATGCTTTTTCGTTCGAAAAAAAAAAAAAAAAAAAATCTTTGCCGCCGCCCCCCCCTAAAAAGGTTTTTTGAAAAGGGGGTGCGTGCAAATAATCATAAATAAAAAAAACCCCCGCAAGTTTCCTTTGTTGGTGTTCGTGCCAAAAATAAAA


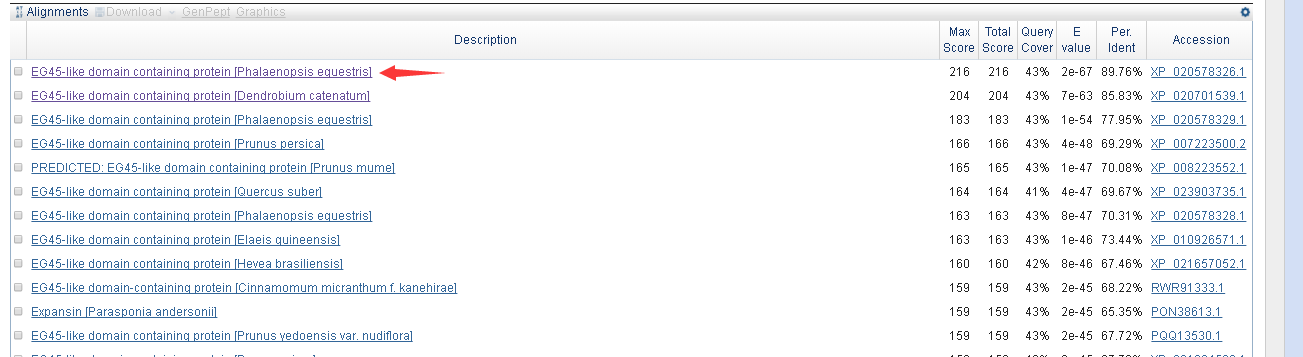


8：

>0605-N56(19030028-10)T7

TCCCTTACCGGACGGTACCAGATTACGCTCACAAGTTTGTACAAAAAACCAAGCAGTGGTATCAACGCAGAGTGGCCATTATGGCCGGGGGTCGTCTTTTTCTTTGGAGAGGCCAGAACTCAACCCTAGAATCTCTCATTTAAATGGCTGGAAGAGGAAAGGCGATCGGATCCGGAGCCTCGAAGAAGGCGACTTCAAGGAGCAGCAAGGCCGGGCTTCAATTTCCTGTCGGCCGTATCGCTCGTTTTCTCAAGGCTGGGAAGTATGCCGACCGTGTTGGCGCCGGTGCTCCGGTCTACCTCGCCGCTGTTCTTGAATATCTTGCTGCTGAGGTCTTGGAGTTGGCTGGGAATGCTGCCAGGGATAATAAGAAGTCGAGGATCGTGCCCAGGCACATTCAATTAGCTGTTAGAAACGACGAGGAGCTCTCTAAGCTCTTAGGCACTGTGACTATTGCTAATGGCGGTGTTATGCCCAACATTCACAACCTTCTCCTTCCTAAGAAGGCTGGTGGTCCTTCTAAATCTGGTCCGGCTGATGAGGAATGAAGTTAGTTGGATTCAAAATCCTAACTTTTTTTTTCCCCTGCTGTTTTATATTTGTTTCTTCCTGTTTAGGATTGTATGTTCTAAATGGATTAGTTTGTACCTCTGTTCATGCTTTGTTGCTGGTGTTGGACAACATGCAGTGAATATATAAAAAAAAATAGCTTTTTTTATATAAAAAAAGAAAAAAAAAAAAAAAAAGAAAAACCGTTGCGGCCCCCCCTCGCCCTCCAAAAGGTTTTTGTACAAAGGGGGGGGGTGCGGGAAAACTCGAAATATCGAAAAAACCCCCAAAAGTCCCTTCATCGTGTCATCGGGCCCCCATATAAAAAA


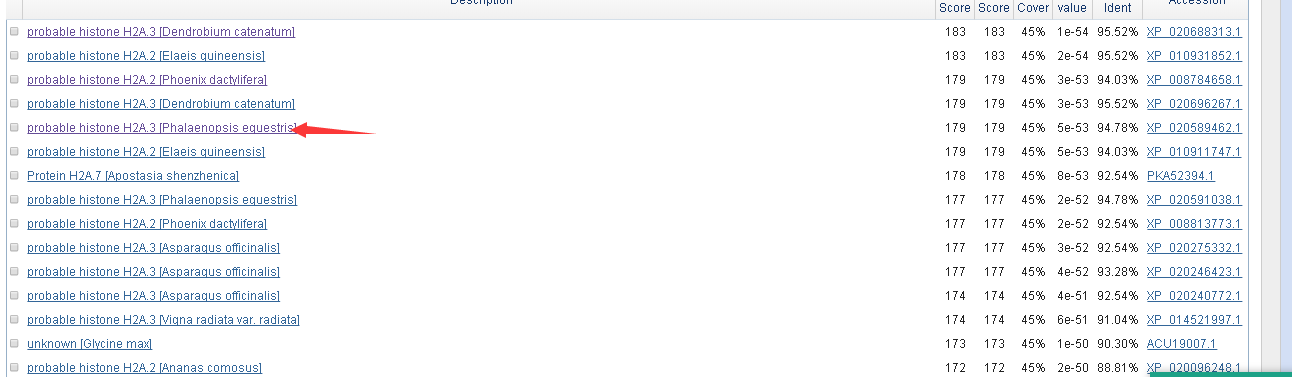

Supplement: Supplementary file 1 — Additional file 1. The sequencing and blast results of the randomly selected eight colonies in library construction. [file 12896_2020_599_MOESM1_ESM.doc]
