# Supplementary material for: Construction and characterization of a high-quality cDNA library of Cymbidium faberi suitable for yeast one- and two-hybrid assays
Source: BMC Biotechnol. 2020 Jan 16;20:4. doi: 10.1186/s12896-020-0599-2 (PMC6966867; doi:10.1186/s12896-020-0599-2)

1：

>0911-19070014-BHLH36-BK-1_T7_C1909110030

CGGCAATCAATACATACGACGTACCAGATTACGCTCACAAGTTTGTACAAAAAACCAAGCAGTGGTATCAACGCAGAGTGGCCATTATGGCCGGGGGCCAAACAGTCGAAGAGCGACCGGGGAAGATCGGCGAGGACTTTTGAATCTGAGGAGACGATGAGGGCAAAGTGGAAGAAGAAGAGAATGAGGAGGCTCAAGAGGAAGCGCAGAAAGATGCGTCAGAGGTCCAAATAATTGGTTGAATGCAAGGAATACTACAATATCTTGCTTTGCTGCTACTAGCTTGTCGAAGAGGATGCTTCGAGAAGGGGAACCTGGGTCCACTACATATTTTTCTGTTATGGGAAATATTTGAACTTAGATTTTAATATAATTTAACTGCTTACTGTGTTGAAAAAATTCTGTTTGTTGACTCTAGTGTGCATCAATGCTTCAGTTGTGGCTGCAAAAAAAAAAAAAAAAAAAAAAAAACTTGGCGCCCCCCCCGCCCTTAAAGGGTTTTTGAAAAAAGGGGGGGTTGAAAAAAAATCAAAAAACCGAAAAACCCCCCAAATTTCCTTTCACTGGGGCTTCGGGCCCCTTATAAA

2：

>0911-19070014-BHLH36-BK-2_T7_C1909110030

GGGGGCCAGTGTGGTCCATACGACGTACCAGATTACGCTCACAAGTTTGTACAAAAAACCAAGCAGTGGTATCAACGCAGAGTGGCCATTATGGCCGGGGGAAGAAAGGAGGTAGATCGGAATCAAGGGCTGCTAGGAGCTTGATCGACGTTGGCACTTCGCAGGATCAATGGGAGTTTTCAGGTTTCATCAGTACCAAGTGGTTGGTCGTGCCCTTCCGACCCCAAGTGATGAGCACCCAAAGATTTATCGCATGAAGCTGTGGGCTACTAATGAAGTTAGGGCAAAATCCAAGTTCTGGTACTTCCTGAGAAAACTAAAGAAGGTGAAGAAAAGCAATGGCCAAGTTCTTGCTATCAACGAGATATTTGAGAAGCATCCGACCAAGATCAAGAACTATGGCATCTGGCTCCGTTACCAGAGCCGGACTGGCTACCACAACATGTACAAGGAGTACAGAGACACCACTCTAAACGGTGGCGTCGAGCAGATGTACAACGAGATGGCCTCCCGACACCGCGTTCGCTTCCCGTGCATTCAGATCATCAAGACGGCGACCATCCCCTCCAAGCTCTGCAAGCGGGAGAGTACGAAGCAGTTCCATGATTCGAAGATCAAGTTCCCTCTTGTCTTTAAGAAGCTTAGGCCGCCATCCAGGAAGCTTAAGACCACCTTCAAGGCATCCAGACCCAATCTCTTCATGTAGAAGAAGTCTTTGATCGGTTTTTTTTTTCATGGAATTTGGCGCTACTAGGTTTTATATGCTCGAGTCTTAATTTTGTTGGATTATTTATCTGATATTGTGACCCTAAACTCCACATTTTTATCTGCTTTTATTGCTCTTGAATCTCTTTGCTACAAAAAAACAGAAAAACAAAACCTTGTGTCGCCCGCGTCCGCCTCCTAGAGAGTTACTGGACAAAGTGGGTGCTGGAGATAGATTCGAAATCCTGGAAAACCCCGGAAGTTCACTTCACACTGTGGCATCGGGCCACACTCAT


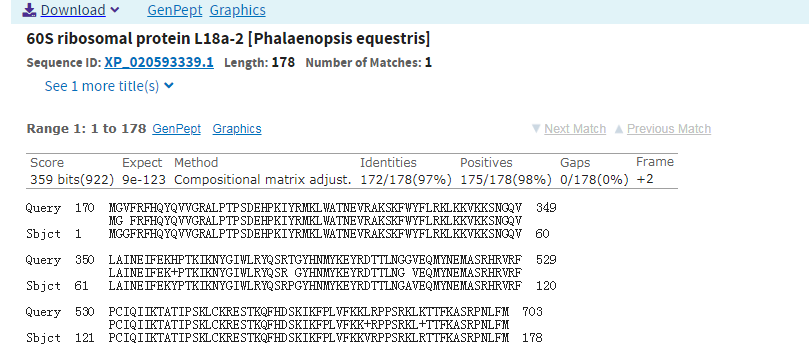


3：

>19070014-BHCH36-2_T7_C1909230036

GTGCCGATGGCGGTACATACGACGTACCAGATTACGCTACACAAGGTTTTGGACACAAAAAACCAGGAGTGGGATTCACGGAAAAGGGGCCTTATGGGCGGGGGAGAGAAGGTGGCTAAAGGAAACTGATAGAAAGGGAGATCGCGATGGGGCGCGGTGGCGACGGAAACACTTCACCTCGTCGCCGGACCAGCAGCAGAGGCGGATCGCCGATCGAGGATATCAAGGTGGAGAAGGCGGAGGGTGTTATTCCTTGGTGGCGGCAGCGGCCGAGCCGGCGGGAGCTCCGTTTAGTGCGGTACGATGAGCTGCCAGAGTATCTTCAGGACAACGAGATTATCCTCAACCACTATCGCGCCGAGTGGACGATCCTTGATGCTCTTCTCAACGTCTTTTCCTGGCATAACGAGACCCTCAATGTTTGGACGCATTTGGGGGGGCTACTCGTGTTCCTTGGGCTCACTGTGGCCGGATCCATAGACATCATTGAGCAGTTCCGCAGCTCCGTCGCTCCCTCCATATCCAGTCTGATGCTTACGCCGATAAAAGTTTCGATTGGGAAGAACTACACGGCGAATGCTATCTCGGATTCCTCCAAAAGCCACCACGTCCCCAGGTGGCCAATTCTCATCTTCCTCACAGGCTCCATGTCTTGCCTTGCCTTCAGCGCCGTCTCCCACCTCCTCGCCTGCCATTCCCGCCGCCTCAGCCTCTTCTTCTGGCGCCTGGACTACGCCGGCATATCCATAATGATCATCTCCTCCTTCTTCCCCCCGATCTACTACGCCTTCCTCTGCCACCCTCTCCCTCGCCTCATCTACCTCTCTTCCATCACCGCCCTCGGCTCCCTCGCTATCGTCACTCTCCTTTCCCCGGGATTCTCCTCCCCTCGCTTCCGCCCCTTCCGCGGCGCTCTATTTCTCGCCATGGGCTTCTCGGGACTTATCCCCGCCGCCCACATCTCGATCGCGTTGGAGCTCGCCATGGCTGCCGCCTATGCGGCTGGCGCGGGCTGTCTACGTTTAGTAGGGTGCCCGGAGCTGTTGGTTTGCCCGGGAGATTTTGGATAATCGCCGGGCAATAGTCATCAGATCTTCCACATGTTTCGTGCTCTTGGCAGCGCCTGACGCACTATGCGACGAATCGATGTGAATGGTGACTGCGCGTGGAGTAACTGGATGCGGTGGGCCGTGGGGCTTGAATGGGGTAAT


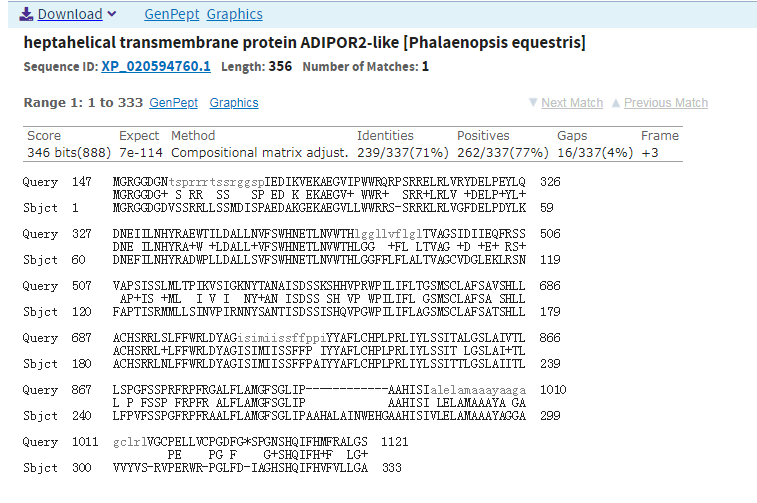


4：

>19070014-BHCH36-2da_T7_C1909230102

GTGCCCATCCGTCATACGACGTACCAGATTACGCTCACAAGTTTGTACAAAAAACCAAGCAGTGGTATCAACGCAGAGTGGCCATTATGGCCGGGGATCCCAGCTTCAGAAAAGCCTCGACTTCCTTATCAATTCCTTTTCCATCGTTTTCCTTAGCAAACCCTAAAAATGTCTTGCTGCGGTGGGAATTGTGGATGTGGCGCCAGCTGCAAGTGCGGCAGCGGATGCAACGGGTGCAATATGTACCCTGGTTTGGCTGAGGACAAGTTCAGCACTTCTGAAACCCTGGTCCTTGGATTAGCACCAACCAAGGGAGGATTTGAGGGATTTGAGATGGCTGAGGGATCTGAGAATGGATGCAAGTGTGGGTCGAACTGCACCTGTGACCCATGCAACTGCAAATGAACTGAAGCTATGTAGATTGAAGGAGGAGACCTCTGCTTGCTTGCTGTTCTAATTGTGATGGTGTGTGCTATCTGCTTATAAAGAATAAGAAGCTTATGGTGATGGAAAAGTATGGTTCCATGAATGAATTCCATGCTCTGCTTGTTTCATGTTGGCAGCTTCTGTTTACTGTGTGTTTTCTTTCTGTGTATCGTTCTGGCTATCATAGTATGATGGAAAATATTTGGCTCTTTCTCCGAAAAAAAAAAAAAAAAAAAAAAAAAAACCTGGGGGCCCCTCCCGCCCTCTAAGGTTTTTTTAAAAAAGGGGGGGCTGCAAATAATTCATAATACCTGAAAACCCCCCAAGTTTCCTTTCAATGGGGCTCGGGCCCCCCATATAAA


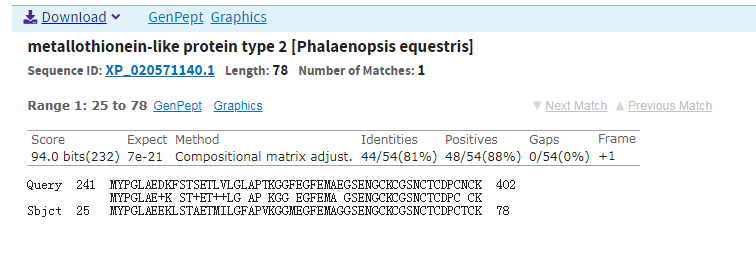


5：

>19070014-BHCH36-3_T7_C1909230036

GAGGCCCATCAATACATACGACGTACCAGATTACGCTCACAAGTTTGTACAAAAAACCAAGCAGTGGTATCAACGCAGAGTGGCCATTATGGCCGGGGATCTAACAGCTAAACGAAGCTTCAAGCTAACTAAAGCAAGCCAGAGAAAAAAAAACTCAGACCAGATATTTTTGGAAGATGAGTTCAAGCTGGTCACTGGAGGTGGAGACCGAAGCCCCAGCGGCTGCTATCTATAAGGCCACTTTCACTGACTGGCACAATCTTGGTCCTAAGCTCCTCCCTGAAATTATCACTAGTGCTACAGTAGTATCAGGTGATGGAAGTCCGGGGAGCATCAGGCAGATCAACTTCTCCCCAGCTGCGCCTTTTAGCTTTGTGAAGGAGCGTCTTGAATTCATCGATCATGAAAAGTTTGAGCTGAAGATTTCTGTTGTGGAGGGTGGACATTTGGGCAGCAAGTTAGAGTCTGCAACTTCACACATCAAGGTCGAGTCAAAGGGAGATGGCTCCGTCGTTAAGCTGACGGCGACATATAAGGCCATCGCCGGCATAGATATTGCCGAGGACACAAAGAAGGCCAAGGAAGGTTTCATCAAGAGCGTCAAGGCAGTGGAGGAATACCTTGCAGCCAACCCAGGAGCCTATGCTTAAAAAATCTTATATTTGCTTGAGTGAGAGTTTGTTTGAAATTGCTATGTGGAAATAATTGGGGTTGGTTTGTGTTCTAAGAGCTTCTATGAGAGTGCTTCGAATATAATTGTTCTATGTTGTGGCTTATTATAAATAAAATGGAAGTATGCTTGCTGCAACAAAAAAAAAAAAAAAAAACTTTTGTGGGCGCCTCTCCGCGCTCTTCAAAAGAGGTCTCTGTGAACAAGGGGGTGGTCTGCAAAAAAAATCCTAAAATCTCTGAAAAACCCCCGAAAGGTTCCTCTTACATTGTGCTGGGGGACCCACCATATTAA


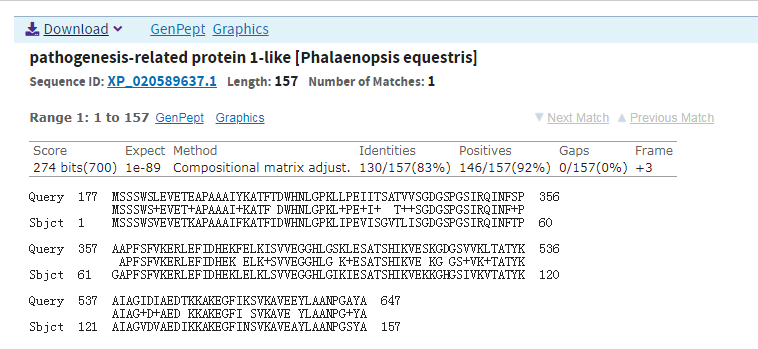


6：

>19070014-BHCH36-4_T7_C1909230036

GTGCCAATCCGTACATACGACGTACCAGATTACGCTACAAGTTTGTACAAAAAACCAAGCAGTGGTATCAACGCAGAGTGGCCATTATGGCCGGGGATCGTTGAGATAAGTTGTTAGTATTCTCTCTCATTTAGAGAAGAATGGAGAAACACTTTGTTCTTGCTCTCACCATTGTTCTTTGCCACTTTGCTCTCTCTTCCGCCATTGATGGAACTGCAACTTACTACACTTCACCTTATACACCTTCTTCATGTTACGGGTATCAAGATATGGGTACGATGATTGCTGCGGCGAGTGATGCTATTTGGGATAATCGTGCGGCGTGCGGACGGAATTATAGGGTCACGTGTACCGGTCCGACCAATCAAGGTGTGCCTCACCCATGTACAGGAGCAAGTGTTGTCGTCAAAATTGTTGATTATTGCCCTGCTGGTTGTCGAGGCACTATAGATCTCTCCCAAGAAGCTTTTGCAGCTATAGCGGATTTAAACGCTGGCAAAATCGAAATTGATTATACTCAGGAGTAGAGGTGACCGGTGGCTGAGGAAAATCGGGCTCTAATATACTTCAAATCTATCAAAATGAGTGCGGAAGTATAGAAGGACTATATCTGTTTCTTAATACATAGAAATAAAGAATCTTCAAATAATAATGGATTATGCGACCAGCATAATAATATAGTTTGTATGGAACGTTGATTGTGCTTCCACTGAGCACAAATGTCTCTCCATCAATAAATATGCTTTTTCATTTTTTTTTTCTAAAAAAAAAAAAAAAAAAAAAAAAAAACCCTGGTGGGCGCCCTCTCTCGCCTCTCTAAAGGTTCTTTTGACAAAAGGGGGGGGCGTGCCAAAGAAACCCAAAATACTCTGAAAAACCCCCCCGAGTCCCCTACCAGTGGTGCATCGGGGGACCCATCATA


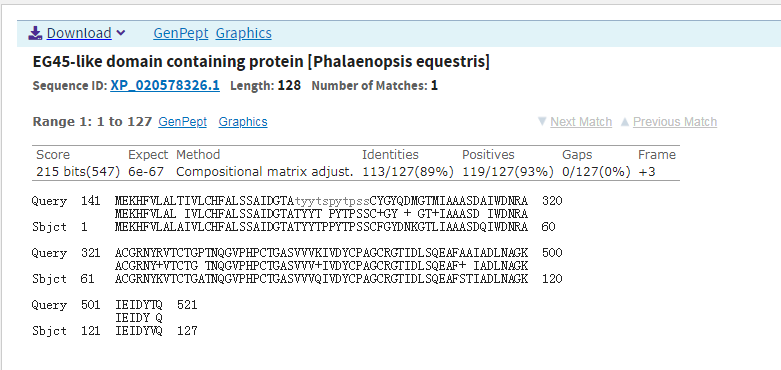


7：

>19070014-BHCH36-5_T7_C1909230036

GTGCCCGTCCATCCATACGACGTACCAGATTACGCTACCAGTTTGGACCAAAAACCCAGCAGTGGTATCCACCCAAAAGGGGCCTTATGGGCGGGAGGGTGAAATTATAGGGAATACTTCTATATCGAGTGAAAAAGCAAGGTGTGCTAGGACGGCTAAGGGTAATTACCCATTGATTTTGAATTCTCGCCTTTGCCGGAACTTGACACTGGTTAACATGGATTAAAAAGCCGTTACTTTGTCTTGGATCTATATTGCTGGAGGAGGAAAGGGTGGCCTTACAACATGTGCTCTGAAATTTAAGGACAGGAGTTTTGAAGAAGAGTTCAGCGGTGTTGTAACAGAACAACCAGGGAAAAAGGGTATTGTGCTGAAGACACCTGAGAATTCTCCTAAAGCTTCTGATGAGCAAAGGGAAGATTTTACTTGATAACTGTTAACCTCTCAATTCCCATATATTCATAAATAAACTCCTGTGATGGTTTAAACCAATATTATCTTTTTTATGCTTGACCTATTGGATGGTGGAATCATTTTGAGTTTTCTATCTGCACTAGTGTATCAATTATTGCTCCATCCTTTTTCCTGAGACTATATCTTACTGAACACTCTTATTCATTTGGAAACCTGGTCATGTATGGATGGTGTTTCTCTTTTATTATTACTTCTGGGATCGATCTTCACTAGATATACTGAATCCATGATTTATACCTTGAGACCATAAATGCTTTTTCATTTTTCGCTATTGGAATTTATTTAAGACAACGACCCCCCCCCCCCCAAAAAGTTTTTTACCCAGCCTCGGCCCTCAAAAGGACTTTAAAAAAGGAAAACCCCCCGAAAGATTCCTTTCAACCTGGAAACCCCCCCCACATTATAATTTTAAATGGGGCATCGGGCCACCCATATA


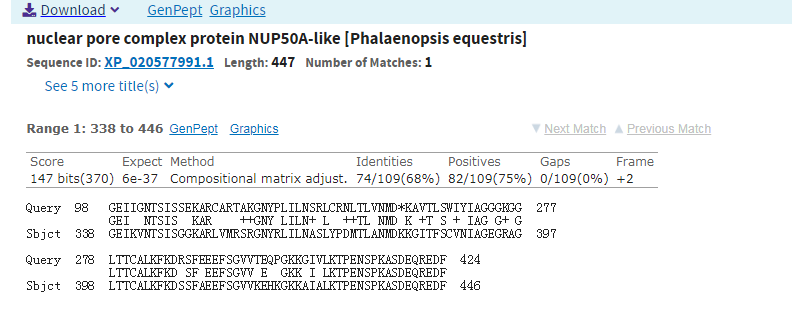


8：

>19070014-BHCH36-6_T7_C1909230036

GTGCCCAGGCGGTACATACGACGTACCAGATTACGCTACAAGTTTGTACAAAAAACCAAGCAGTGGTATCAACGCAGAGTGGCCATTATGGCCGGGGATCCATCAACACTTGAGCTACATAACCCTAAGTTTCAGCCTCACCGTTTAGCTCTCTAATGGCGACCTCTGCGATTCAAGGCTCAGCCTTCGCCGGCCAGACTGCTCTCAAGCAGTCTAATGAACTCCTTCGGAAGGTCGGTTCCATCGGCAATGGCGGCCGCATCACCATGCGGCGGACTGTTAAGAGTGTTCCGCGGAGTATCTGGTACGGCCCAGATAGGCCCAAATACCTGGGCCCCTTCTCGGAGCAAACGCCCTCCTATCTGACAGGAGAATTCCCCGGCGACTACGGCTGGGACACCGCGGGGCTCTCCGCGGACCCCGAAACCTTCGCCAAGAACCGGGAGCTGGAGGTGATCCATAGCAGATGGGCCATGCTCGGCGCACTGGGCTGCGTCTTCCCCGAGCTCCTCGCCAAAAACGGCGTCAAGTTCGGCGAAGCCGTTTGGTTCAAGGCCGGGGCCCAAATCTTCTCGGAAGGAGGCCTGGACTACCTCGGCAACCCTAACCTGATCCACGCCCAGAGCATCCTAGCCATATGGGCCACCCAAGTCGTCCTCATGGGCTTCGTCGAAGGATATCGGATTGGTGGCGGCCCGCTTGGCGAGGGACTCGACAAGATTTATCCTGGAGGGGCCTTCGACCCATTAGGCCTTGCGGATGACCCGGAGGCCTTCGCGGAGCTGAAAGTGAAGGAGTTAAAGAACGGGCGGCTGGCCATGTTTTCCATGTTCGGGTTTTTTGTGCAGGCTATTGTGACCGGGAAAGGGACCCATTGAGAACCTGTATGACCACCTGGCTGACCCGACTGTCATAATGCTTGGCTTATGCCACCAACTTTGTGCCTGGAATGAGTGTGTCATTGATTGTCTGATGTTTTCACTCATATGGAATGGACCATTTCCTCCACATACAACATACTGACCAGAAACTCTTGGTGCGCCCTGCTCTCGCCCTTAAGAGGTTCCTTGGACCAAAGGGGGTGGGTCTGCAGTGTATTCGTATATCTGAACCTCGGAGTTCTCACTGGGATGGGGGGACCATATCTATACCTGAGCTG


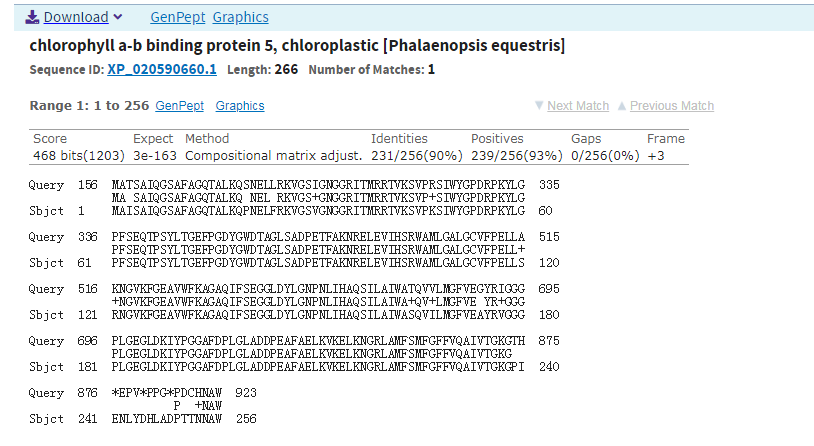


9：

>19070014-BHCH36-7_T7_C1909230036

TTGCCATCCATACATACGACGTACCAGATTACGCTACAAGTTTGTACAAAAAACCAAGCAGTGGTATCAACGCAGAGTGGCCATTATGGCCGGGTCTGCAATCCAATTATCGCGAAGATGTATCAGGGAGGTGGGGATGTGCCTGCTGGTATGGATGAAGAGGGTCCTGCTCCTGGCGGTAGTGGCGCTGGTCCTAAGATTGAGGAGGTTGACTAAGGGTTTTCTTCTTATAAGTAAAGCTAATGTTATACAGGGATACGCTCTGCTGTTATTTTAAGTGTTTTATTTTGCTGTAATGAAGTTTGGACGTTCGTACTTTTTATGTTGAATAAAATGGTTTTGTAGCGTTGAGATAAAAAAAAAAAAAAAAAAAAAAAAAAAAAAAACCTTGCCGCCCCCCCCCCCCCCAAAGGGTTTTTGAAAAAAGGGGGGGTGGAAAAAAAATCAAAAAACCGAAAAACCCCCCAAGTTTCCTTTCATTGGGGCTTCGGGCCCCATAAAA


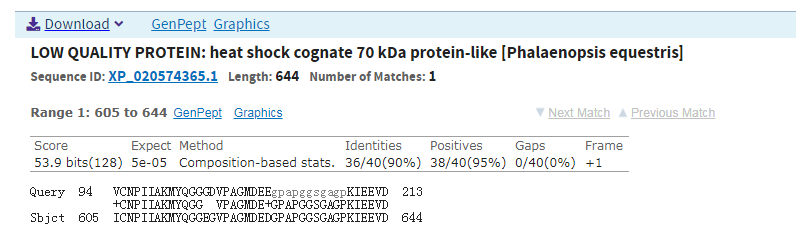


10：

>19070014-BHCH36-8_T7_C1909230036

TAGCCATCGTACATACGACGTACCAGATTACGCTACAAGTTTGTACAAAAAACCAAGCAGTGGTATCAACGCAGAGTGGCCATTATGGCCGGGGATCCCAGCTTCAGAAAAGCCTCGACTTCCTTATCAATTCCTTTTCCATCGTTTTCCTTAGCAAACCCTAAAAATGTCTTGCTGCGGTGGGAATTGTGGATGTGGCGCCAGCTGCAAGTGCGGCAGCGGATGCAACGGGTGCAATATGTACCCTGGTTTGGCTGAGGAGAAGTTCAGCACTTCTGAAACCCTGGTCCTTGGATTAGCACCAACCAAGGGAGGATTTGAGGGATTTGAGATGGCTGAGGGATCTGAGAATGGATGCAAGTGTGGGTCGAACTGCACCTGTGACCCATGCAACTGCAAATGAACTGAAGCTATGTAGATTGAAGGAGGAGACCTCTGCTTGCTTGCTGTTCTAATTGTGATGGTGTGTGCTATCTGCTTATAAAGAATAAGAAGCTTATGGTGATGGAAAAGTATGGTTCCATGAATGAATTCCATGCTCTGCTTGTTTCATGTTGGCAGCTTCTGTTTACTGTGTGTTTTCTTTCTGTGTATCGTTCTGGCTATCATAGTATGATGGAAAATATTTGGCTCTTACAAAAAAAAAAAAAAAAAAAAAAAAAAAAACTTTGGGGCCCCCTCCGCCCTCAAGAGGTTTTGGAAAAAAGGGGGGGGGGAAAATAATTCAAAATTCCGAAAAACCCCCGAAGTTCCCCTTCACTGGGGCTCGGGGCCCCCCTTAAAAA


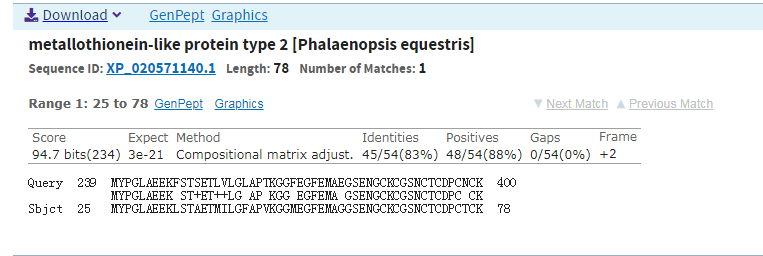


11：

>19070014-BHCH36-9_T7_C1909230036

GAGCCAATCGGTACATACGACGTACCAGATTACGCTACAAGTTTGTACAAAAAACCAAGCAGTGGTATCAACGCAGAGTGGCCATTATGGCCGGGGAAGTAAATCAATACCCAAACTCCCAACTAATCAACACAGCCATGGCCTCCTCCACCTGCTCAGCAGCAGCCCTCCTCCTCCTCTCCGCGGCAGCGCTGTTGAGCCTCCTAACCACTCCCGTCTCCGCCGACGACCGCCTGAACGCAGGCCAGTCCCTTGAGGGGGGACAATCCCTCGCACAAGGCCCCTACTTGTTCACCATGCAGCAGGATTGCAACCTCGTCCTCTATGACAACAACGGGGCCATCTGGGCGACGGGGACCAACGGAAAAGCCTCCGGCTGCGTCGTCACGATGCAGACCGACGGCAACCTCGTCATTTATAGTGGCAGCAGTGTTATCTGGGCAAGCAACACCAACCGCCAGAATGATAACTACTATCTCATCCTCCAGAGAGATCGCAACGTCGTCATCTACGACAGCTCCAACAACGCTATTTGGGCGACTGGCACCAACGTCGGCAATGCTGCTGTTGTCGTCATCCCTCACAGCAACGGCACGGCGGCTGCGTCTGGCGCTGCGCAGAACAAGCTCAAGGAACTGTATCCATGAAGGCTAATATCACAGAGAGGAAGTATTCTTAATATATTTTGAATAAATTGGCGATGACAACTGTGGTGCTATTGCCTAACGCAAGCTAATTTTCCTTTTTATAACTGTGTTGTCGTAGTATGAATGATCGTATGAAATAAAGTTGCTTGTTTAAATTATCATATATGGAATTCGTTTGGGCCTAAAAAAAAAAAAACAAAACCCTGGGGGGCGCCCTCCTCGCGCCTTCCATAAGAGGTCTCTGTGACACAAAAGTGGGTGGGCGTGGCAGAATGAATTCGTAAATATACTAGAAAAACCCGCGCAAGGTTCACTCTTCACATGGTGGCATCGGGGGGCACAACACTATTA


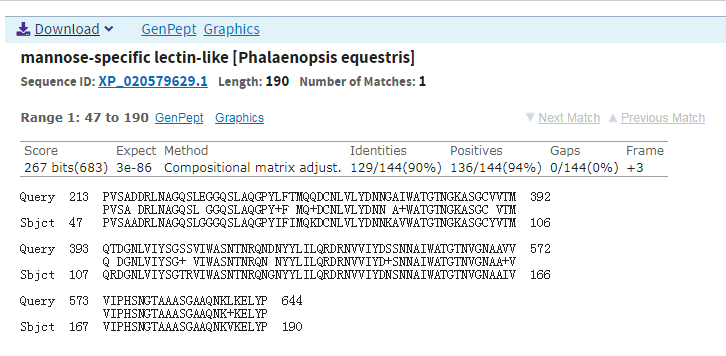


12：

>19070014-BHCH36-10_T7_C1909230036

GAAGGAAATGCGTCATACGACGTACCAGATTACGCTCACAAGTTTGTACAAAAAACCAAGCAGTGGTATCAACGCAGAGTGGCCATTATGGCCGGGGGACCCAGAGATATCTTACATTTCTCCAAAAAAGTAAAAACACATTGGGCCTGCTCTCACCGTTGTTCTTTGCCCCTTTGCTCTCTCTTCCGGCATTGGTGGATGTGCCTCTTACCCCCCTCCGCCTCATACACCTACTGCATGTTACGGGGATCAAGATATGGGGGGGAACATTGCCGCGGAGAGTGATGCTATTTGGGATAATCGTGCGGGATGGCGACCGCATTATCTGTGACCGCGACCCGGCCCTATGGATCACGGTGACCCTCACCCATGTCCCCGACTGCGTGTTGACGACAAAATTGTTGATTATTGCCCTGCTGGATGGCGAGGCACTAGCTATCTGTCCCAAGAACATCTTGCACCTAAATGATATTTACTATCTCATCCTCCCGAAATATCTTATACTCATCATCTAAGATGACTGGTGGCTGATATTAGGGCGACTCTAACCAACTTCAAATCTATCTGCTGAAGTGCGGACCCTAGAAGGACTATATCTTTTTCTTAATACGTAGCTGTACAGAATCTTCTCATAGTACTGTATTATGCGACCATAATATCACATATAAGGGATTATAGAACTTTGATTGTGCTTACACTGACCACAAATGTCTGCGCCATCTATTGCCTATGCTTTTTTATTTTTCCTCATTATAACTGTGTTGTCGCAATATGAATGATCGCATGCAATACAGTGGCTTGTTTAAAATATCGGAGGCTCGAATTGAAAGCTATAAACAGAAAATGTCGGGAATTTGACTTCTATTGGTTCTCCGGTCGCACCATGTATAGGGCGTCATAGAATCGTAGGATGCTTGAAAAAACCGCCCCCGAGTTATCCTCTGCAACAGGTCGCGTCGGGGACACTCATAAATGG


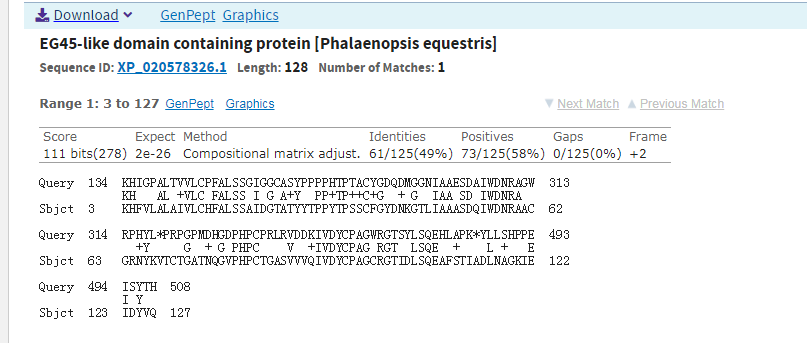


13：

>19070014-BHCH36-12_T7_C1909230036

TAAGCCCATCCATACATACGACGTACCAGATTACGCTACAAGTTTGTACAAAAAACCAAGCAGTGGTATCAACGCAGAGTGGCCATTATGGCCGGGGATCGTTTAGATAATTTGTTAGTATTCTCTCGCATTTAGAGAAGAATGGAGAAACACTTTGTTCTTGCTCTCACCATTGTTCTTTGCCACTTTGCTCTCTCTTCCGCCATTGATGGAACTGCAACTTACTACACTTCACCTTATACACCTTCTTCATGTTACGGGCCCCTTTATTCGGCTACGATGATTGCTGCGGCGAGTGATGCTATTTGGGATAATCGAGCTGTCTGCCCACGGAATGATCAGGGCACCTGTACCGGTCCGACCCAACATTGTGAGCCTCACCCATGTTCCTGATCAAAAGATGACCATGACATTGACTATTATTGTCCTGGAGGAAGCCGAGGCACTATCCATCTCTCCCGAAAAGATTTTGCTATTACTGCGAATTAAAGCACTGGCAAAATCACTGTTGATTATTCTCTCGAATAAAAGGGACCATGGGCTGAGGAAAAACGGGCTCTAATATACTTTTTATCTATTTTTCTGATTTATTTTGCTATAGAAGGACTATATCTGTTTCTTAATACATAACCCTAGATAATCGTCATATAATAATGGATTATGCCACCAGCATAATAATATAGTGATGTATGGAACGTTGATTGTGCTTCCACTGAGCACAAATGTCTCTCCATCAATAAATATGCTTTTTCATATTTTGAAAAAAAAGGGGGGTTAAAAATTTGTCGGCCCCCCCGAAAATCCCGGGGTTTTTGCCCAAAGGGGGGGTTGGGGAAAAAATTTAAAAATACTGAAAAACCCCCCAGGTTCATTTCACTTGGGCATGGGGCCCCCCTATAA


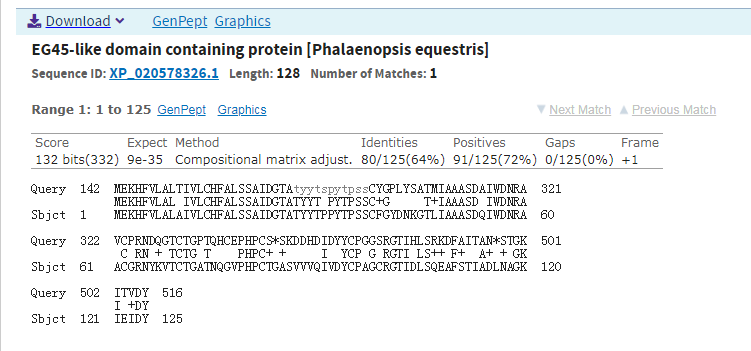


14：

>19070014-BHCH36-13_T7_C1909230036

GTGGGAATTCCATCATACGACGTACCAGATTACGCTCACAAGTTTGTACAAAAAACCAAGCAGTGGTATCAACGCAGAGTGGCCATTATGGCCGGGGGCAGTGCAGCAGCTTAGCTCCGCTCAAAGTTCTTCATTTGGAAGGAGAAAGGAAGGGCGAAAAGTATGGCTTCTTCAATGATGGTCTCGTCCGCCACTGTGGCGGTCGCTCGATCGACGGCTCCGGCTCAGTCGACCATGGTCGCTCCCTACACCGGTCTGAAGTCTGGTGCTATTTTACCCGCCGCCCGTAGAGCCGCCACGAACCTCAACCACCTCCCGAGCAATGGCGGCAGAGTCCAGTGTATGAAGGTGTGGCCTATTGAGGGTGTGAAGAAGTTTGAGACACTATCTTACCTTCCACCGCTTAGTCCAGAGGCTTTGCTCAAGCAGATTGATTACCTTCTCCGTTCGGGATGGATTCCATGCTTGGAGTTCTGCAAGATTGGATTCGTGTATAGGGAGAATCACAAGTCACCTGGATATTATGATGGACGGTACTGGACGATGTGGAAGCTACCAATGTTCGGATGCAACGATGCAGTTCAAGTGGCGAAAGAGGTGGAAGAGTGCAAAAAATCATATCCTGACGCTTTTATTCGAATTATTGGATTTGATAATGTGCGTCAAGTACAATGCATCAGTTTCATCGCCTATAAGCCTCCAGGCAAGGACTACTACTAAGATGTCCTAAGAAAGCAACTAATGCTCTAGAATATTGTTGTAGTTTTCTATTATTGTCCATGGATTTCTCCATATTTTTTTTTGAGGGTTTAAGACCTTTTGTTCATGTTGATTTCCTATGTTGCTTTTGTTTATCTCTGTCATGCTCTTCTGAATGGTGGGAACTTAGAAAGCAATTTGACCCAGAAAAAAAAAAAAAACCTTGTGCGGGCGCCGCTCTGCGCGCCTCTACTAGAGAGGTTCTCTTGTATCACAAGGGTGGGTGGGTCTGGCAGATTGAATCTCGTAAAATTCACTGAAAAAACCTCGCGCAAGTTCACTCTCCACCTGTGTGCACGTGGGGCGCCACACTATATATATAA


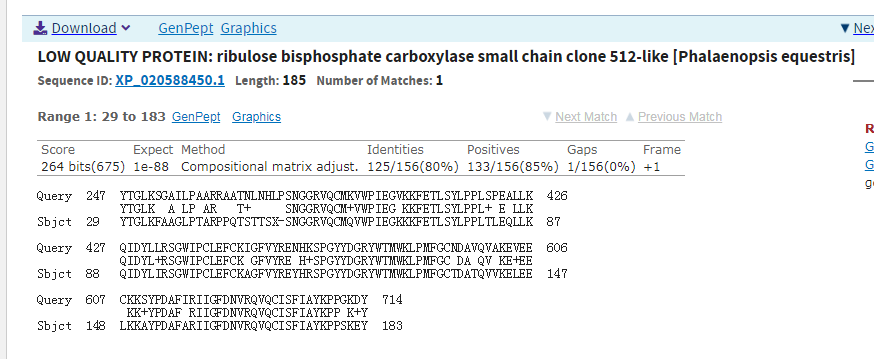


15：

>19070014-BHCH36-14_T7_C1909230036

GAGGGCCGGTGCGTCCATACGACGTACCAGATTACGCTACAAGTTTGTACAAAAAACCAAGCAGTGGTATCAACGCAGAGTGGCCATTATGGCCGGGGGTTTCTAAGCGCCATCTCCCTTAAAGAGTTTCTACTATCTCTCCTCCTCCTACTATGCGGCCTTTCTCCCCTCACCCTCAAACTCTCCGGCGGCGCTACCTCTCTCCACATGTGGGCCCCTCGCCGCCGCCGCCGACGCCGCCATTCCCGTCCCGCCCTAGTCCTCCTCCACGGTTTTGGAGGATTCGCCAAGTGGCAGTTTGAGGGCCAAATAGTGCCGCTTTCTCGCCACTTCGATCTCTACTTGCCGGACCTCATCTTCTTCGGTCGGTCAGAGTCGGCGGAGGGGAAACGGTCGGTGAAGTTTCAAGCCAAGTGTGTGGCAGACGCTATGAGAAAGCTTGGGGTGGAGAAGTATGCGGTGGCTGGGGTGAGCTATGGGGGCTTTGTAGCGTATTCTATGGCGTCGGCGGAGGCGGAGGCGGAGGTGGAGAGGGTTGTGGTGATTGCATCAGGGGTGGCAGCCACGGCGGAGGAGAGAAAGGAGTTAGCGGAGAAAGAGGGGAGAGATGTTGCGGAGTTATTGCTGCCGCAGAGAGTTGAGGATTTGAGAAATTTGTTTCGTAGGTGTATTTTCAGACCGCCGACTTGGATGCCCGATTTTGTTCTTCGTGATTTCATTGAGATAATGTTCAAAGAACACAGAAAGGAGAGGGCAGAGTTGCTGAATGAACTTCTATCGAATGGAATTGGATGGGGATCTCTTCCCGTTCTTAAGCAAGAAACTCTAATCTTATGGGGCGAGAGAGACGCTATATTTCCTTTGCCTCTTGCTTATGCTCTTAAGAGACATTTGGGAGACAAGGCAAAATTACAAGTGATTAAAGGAAGCAGGGCATGCATTACTCATTAGAGAAGCCTCATCATGTAAATAGTCTGATTAGGAGGTTCGTTTTGCATGAAAAAATGGTTTAAAGTAAGTGAGAAATCATATATACTAATATACGAAATAGACAAGTCTCGACTGATTAGTCATATGGAGGATGGAACAGATGATCGACTTCAAGTTACGGAGCCTCCCATCGAAGAATATTTGAGCTTAAATACCTGCATCATGGTGATTACTCACCACTCAGAAAATAATTTGGGAGTTATTGTGTGAATTCGCGCAGA


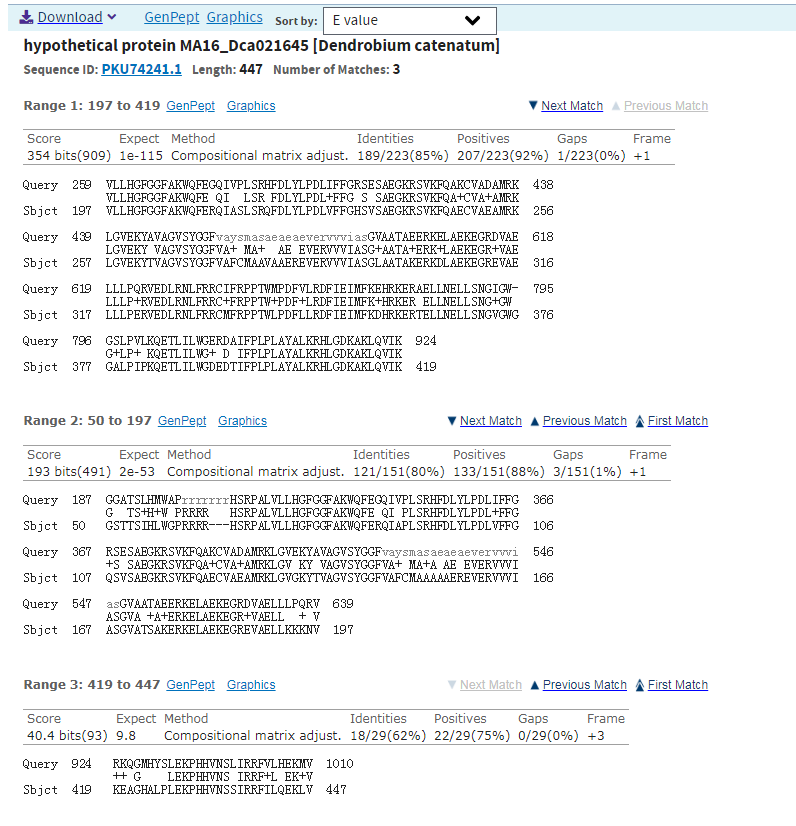


16：

>19070014-BHCH36-15_T7_C1909230036

TAGGGCAGGCATACATACGACGTACCAGATTACGCTACAAGTTTGTACAAAAAACCAAGCAGTGGTATCAACGCAGAGTGGCCATTATGGCCGGGGAGAGCCGGATTGTAAACCCTAGACGAGCAGCCGCCGCAGCGCCAAAGAGCTCCTGGACGAAAAATGGGCATCGATCTCATTGCCGGAGGGCGGAGCAAGAAGACGAAGCGCACCGCGCCCAAGAGCGACGATGTTTATCTCAAGCTTCTCGTCAAGCTGTACAGGTTTCTTGTAAGGAGGACGGGTAGCAAGTTCAATGCTGTTATCCTTAAGAGGCTCTTCATGAGTAAGATCAATAAGCCTCCTCTCTCTCTTAGGAGACTTGCAAAATTCATGAATGGAAAGGAAAATAATATTGCGGTTATTGTCGGAACTGTAACCGACGATAAGAGGGTGCACGAAATTCCAGCCATCAAGGTGACGGCTTTGAGGTTCACAGAGACTGCTAGGGCAAGGATCCTTAAGGCCGGAGGAGAATGCCTGACCTTCGACCAGCTTGCTCTTAGATCGCCTCTTGGACAAAACACGGTTCTTTTACGGGGCCCTAAGAATGCTAGGGAAGCTGTGAAACACTTTGGGCCTGCACCTGGTGTTCCGCACAGCCACACAAAACCCTATGTTCGTTCCAAGGGAAGAAAGTTTGAGAAGGCAAGGGGCAGAAGAAATAGCCGTGGTTTCCGGGTCTGAGCTTGTTCTTCTTGTTTAGGTTTCTGAATTTGAAATCAAACAGTTCTTCTCTATTAGTAAACAGTCTTTTTGGCAGTTTTTAACACATTTGAGTTGTATTAGTAGTATATTTTTGATGAATCTTTTGGTTTTGAACAAACTTGCCATCACCCTTCTGTTTTTGTTTTGAATCGAAACATCTCTGTTAGTACCAAAAAAAAAAAAAAATAAAAACTTGTGTGTCGCGGCCGCGCTCTCGGCTCCTTAGAGGTTCTCTGTGTGACAAAAAAGGGGTGCTGCTGCAAGTAGTAATCTCGAAATATCTCTGAGAAAAAACCCCGCAGAGGTCACCCTCCAACTGGCCGTCTGGGGCGCACACTACATATAAAAAA


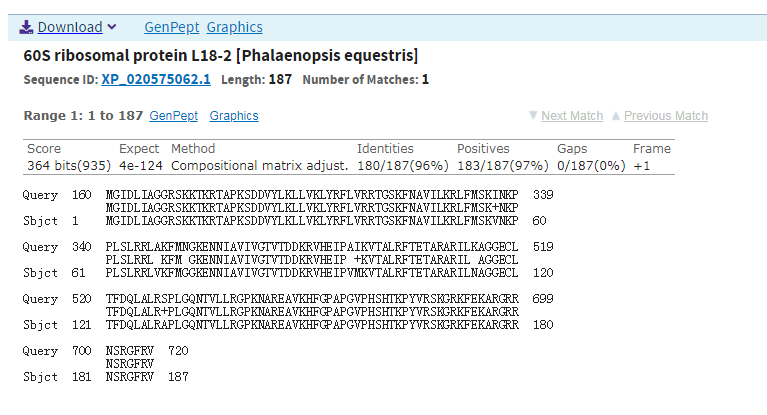


17：

>19070014-BHCH36-16_T7_C1909230036

GACCCAATCCGTACATACGACGTACCAGATTACGCTCACAAGTTTGTACAAAAAACCAAGCAGTGGTATCAACGCAGAGTGGCCATTATGGCCGGGGACCCACCGTTCCAAAGCCAGCCGTCCCCGGCCCGCCGTCGAAATCGTCGGATTCAATGGACCGCTGCTGCTCTATTTGTGTTTGTCTCTTATCTCTCTTCTTCCTGCATCTTGTTCCGTTGGACGGTCGCTTGGATCCGGTCCTCCGGTTACCTTCCGAAATCTCCAGCCGCCGCCTGGAAGCCGGCGGAGATGATTCAGTTGGGACGAGATGGGCCATCCTGATTGCAGGATCGTCTGGCTACGGGAATTACCGGCATCAGGCTGATATATGCCATGCTTACCAAATCATGAAAAATGGGGGTCTTAGAGATGAAAATATCATTGTTTTCATGTATGATGATATTGCATACAATGAGGAAAATCCTAGACAAGGAATTATAATAAACCGTCCTGATGGTGGTGATGTTTACGCTGGAGTTCCCAAGGATTATGTAGGAGATGATGTTAATGTTGACAATTTTTTCGCTGTTCTCCTTGGAGACAAAAAGGCTGTATCTGGTGGTAGTGGGAAAGTTGTTGACAGTGGACCTGATGACCATATTTTCATATTTTATTCAGACCATGGTGGCCCAGGAGTTCTAGGCATGCCTACATATCCTTACCTTTATGCGGATGATTTCATCTCTGTTTTGAAGAAAAAGCATGCCTCCAATTCCTACAAAAGCTTGGTCATCTATCTTGAGGCTTGTGAATCTGGTAGTATTTTTGAGGGTCTTCTCCCTGAAGATATAAATATATACGCAACAACAGCATCTAATGCTGTGGAGAGTAGTTGGGGAACTTATTGTCCTGGAGAAAGTCTTAGTCCACCTCCAGAATATTGGACATGTTTGGGAGACCTCTACAGCATATCTTGGATGGGAAGATAGCGACATTCATAATCTGAGGACTGAAACTCTGAAGCAGCAGTACAAACTAGTCAGACAGGACATCAGTTGATAGCTCATATAGCCAGTTCTCATGTCATGCAATATGGATCAGATGATCTCACAGAAAATGTTTTCTGTACATGGGTCAATCTAGCACGATATTCTACCTTCAATGAGGATACTTCTTCTTCATTTAACACCTAACGCA


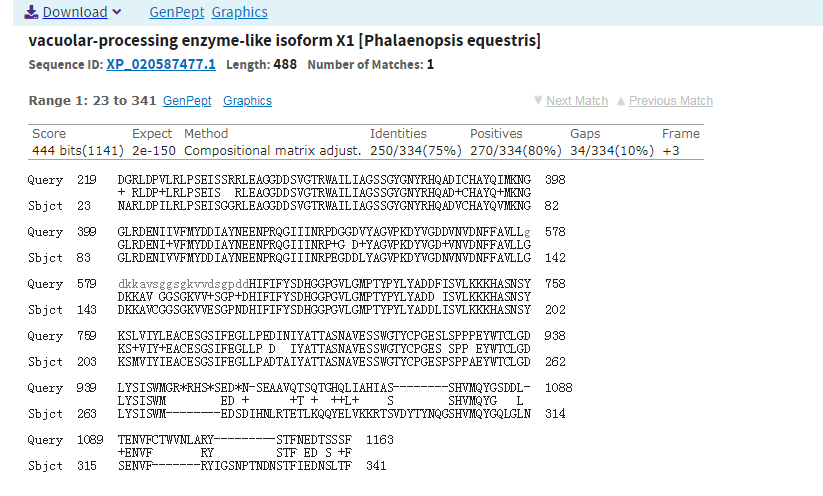


18：

>19070014-BHCH36-17_T7_C1909230036

TAGGCGCCGTCCATACGACGTACCAGATTACGCTACAAGTTTGTACAAAAAACCAAGCAGTGGTATCAACGCAGAGTGGCCATTATGGGCGGGGAAGTAAATCAATACCCAAACTCCCAACTAATCAACACAGCCATGGGCTCCTCCACCTGCTCAGCAGCAGCCCTCCTCCTCCTCTCCGCGGCAGCGGTGTTGAGCCTCCTAACCACTCCCGTCTCCGCCGACGACCGTCTGAACGCAGGCCAGTCCCTTGAGGGGGGACAATCCCTCGCACAAGGCCCCTACTTGTTCACCATGCAGCAGGATTGCAACCTCGTCCTCTATGACAACAACGGGGCCATCTGGGCGACGGGGACCAACGGAAAAGCCTCCGGCTGCGTCGTCACGATGCAGACCGACGGCAACCTCGTCATTTATAGTGGCAGCAGTGTTATCTGGGCAAGCAACACCAACCGCCAGAATGATAACTACTATCTCATCCTCCAGAGAGATCGCAACGGCGTCATCTACGACAGCTCCAACAACGCTATTTGGGCGACTGGCACCAACGTCGGCAATGCTGCTGTTGTCGTCATCCCTCACAGCAACGGCACGGCGGCTGCGTCTGGCGCTGCGCAGAACAAGCTCAAGGAACTGTATCCATGAAGGCTAATATCACAGAGAGGAAGTATTCTTAATATATTTTGAATAAATTGGCGATGACAACTGTGGTGCTATTGCCTAACGCAAGCTAATTTTCCTTTTTATAACTGTGTTGTCGTAGTATGAATGATCGTATGAAATAAAGTTGCTTGTCGAATTCAAAAAAAGAAAAAGGAAAAAAAAAATGTGGCGGGCCGCCCCGCCCCTTAAAGTTTTTGTGAACAAAGGGGGGGGGCGTGGGAGAGAATTCATAATATACGAGAAAAACCCCCCGCAGGTTCCCCTCCATGTGGGGCATGGGGGGGCGCCCAATATATATAAAAAA


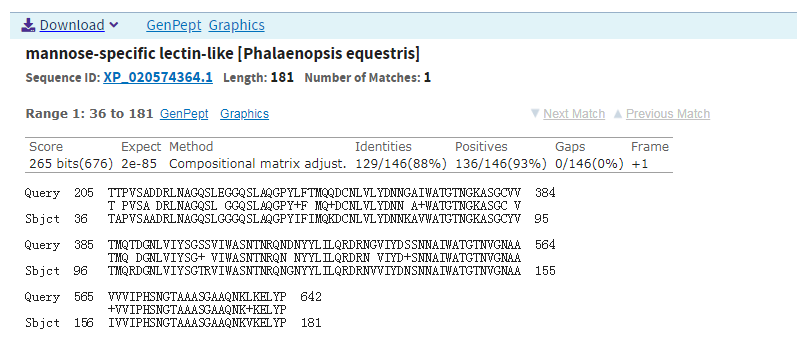


19：

>19070014-BHCH36-19_T7_C1909230036

CATGCCAGCCATCCATACGACGTACCAGATTACGCTACAAGTTTGTACAAAAAACCAAGCAGTGGTATCAACGCAGAGTGGCCATTATGGGCGGGGGAAGGATAGAGCCATGTATCGCCAAGGCTCTCGCTTACTGATACGCGCAACCGCCGCCGCCAGAGTCCGCTCCTCGGCGGCTACCGGCAGCCGAGCCGCTTCCCAGGCTTTCTCCACCGAAGTTACGGAGGCGCCGGCGGTAGATTCGACGTTCCTTGAGGCATGGAAGAAGGCAGCCCCCAACATCGACCCCCCGAAGACTCCGTTAGCATTTATGAAGCCGCGGCCGCCAACTCCTTCCTCCATCCCCTCCAAGCTGACCGTCAACTTTGTTGTCCCTTACCAGTCCGAGATCTCCAACAAGGAGGTTGATATGGTGATTATACCTGCAACAACTGGGCAAATGGGTGTTCTGCCGGGCCATGTGGCAACCATAGCAGAACTCAAGCCTGGTCTTCTATCGGTGCACGACGGAAACGACGTCACCAAGTACTTTGTTAGCAGCGGCTTTGCATTCATCCATGCAAACTCTTACGCAGATATCGTCGCCGTGGAGGCTGTTCCCGTCGACCAAATAGATTTGAGCCTGGTTCAAAAAGGCCTAGCTCATTTCACTCAGAAGCTTAACTCCGCCGGCACCGACCTGGAAAAGGCAGAAGCTCAAGTTGGAGTCGATGTACACAGCGCACTCAATGCCGCACTTACTGGCTGAAACCAGACTCTCTTCTTTGAACGAGAATCCAAATGGATTCAATTGAGAGTGCCCGTAGAATTGGAATAATTTGATCCTGAATTTGAAATGTTTATTTCATGTTATTGGTTACAAATTTGCTTCGTGTTTGTGTATCATCAAAAAACAAAATTTTATTTTCAATACGTTCTGAGAGCTAAATGTCTACTATGAACGGCTGTATAATACTGGCACAGTCTGGCACTCTGAATCCTTTCAGTATTCAGTCTTATTTGTGAAGCCGAGGACACTACTACTACTTAAACTCCTTGTCCGGTGGCTTAGGCTTTAAGAGGCTCTCTTGGAACAAGTGGGGCTGCCAATGATCCGAAATACTGAAACCCGGAAGTTCACTTCACACTGTGACTGGGGGGAACC


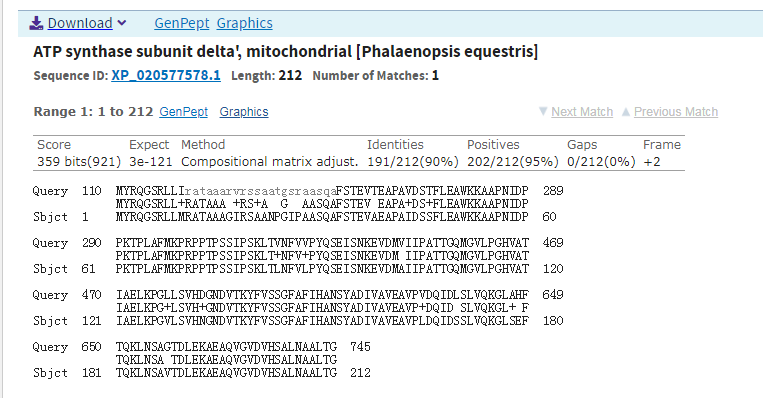


20：

>19070014-BHCH36-20_T7_C1909230036

GGCCCCATCCGTACATACGACGTACCAGATTACGCTCACAAGTTTGTACAAAAAACCAAGCAGTGGTATCAACGCAGAGTGGCCATTATGGCCGGGCTCCGCTACGGACTTCGATCTCTCGCTTCTTCGTTCTGATTTTCTCCATGGTTGCTTGCTTTGATCTTAGTCCCTCATTTCTATAGCAATCAGCTGGAGAAAGCGATGGTATTTACTTTGTTCTAGAGTAGAATCGCCGGTCATTGGTGAAACCCTAGTAGGAACCTACTTTCTCTCTAAAATTCGGCTTGCAGGTTGTGAGAATGTCGACTCCTTCAAGGAAGAGATTGATGAGGGACTTCAAACGATTGCAACAAGACCCACCTGCTGGCATCAGTGGCGCCCCTCATGATAATAATATTATGCTCTGGAATGCGGTCATATTTGGCCCAGATGACACCCCTTGGGATGGAGGTACGTTCAAGTTGACACTTCAATTTACTGAGGATTATCCAAATAAGCCACCAACTGTGCGGTTTGTTTCACGAATGTTCCATCCAAATATTTATGCAGATGGAAGTATTTGTCTGGATATTCTGCAGAATCAGTGGAGCCCAATATATGATGTAGCAGCAATTCTTACATCAATCCAGTCATTGCTGTGTGATCCAAACCCCAACTCACCTGCAAACTCAGAAGCTGCTCGGATGTTCAGTGAGAACAAGAGGGAGTACAACAGGCGAGTGAGGGAGATTGTCGAGCAAAGCTGGACAGCAGATTGAACAAACTATCACTAAATAAAACAAGAAGAAGAAGAAAAGAAGAAAATGGTTTGGTGCTTCTTCTTCCCTCTCATCATTCCCATCTAAAAAAAAAGAACTCTTAGGTTTTATTTGCTGAATGAAATGATTGAACCTTTTGAAATATTATGTACTCCACAAGAAACTGCTACCTCCTGTACTGTATATTGGCTTGATCGGAAAGATAAAGAAGAAACCCTGTGCGCGGCCCGCCCTCGCGCCCTCTCATAAGAGGTCTCTTGGTACCAAGTGGGTGGGCGTGGCGAGGTGTGAATTCTGTAAATATTCTTGAAAACTCGGAGGTTCACTCTCCACTGTGCACTGGTGGGCCACACACTACTAT


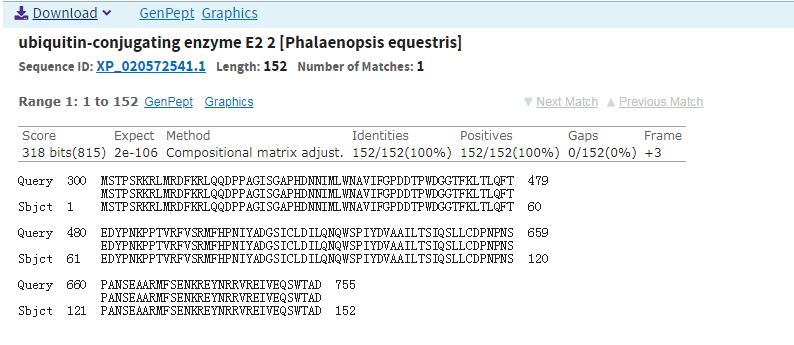

Supplement: Supplementary file 2 — Additional file 2. The sequencing and blast results of the 20 selected positive colonies in library screening. [file 12896_2020_599_MOESM2_ESM.doc]
